# Unraveling Bone‐Skin Crosstalk Enables miRNA Nanoformulation for Cutaneous Neurovascular Reconstruction in Diabetic Mice

**DOI:** 10.1002/advs.76500

**Published:** 2026-07-11

**Authors:** Tao Shen, Hongdou Shen, Weiyue Wu, Zhuoying Jiang, Bin Liu, Yansi Xian, Rui Peng, An Yan, Yu Ben, Yong Shi, Xiang Chen, Baosheng Guo, Qing Jiang, Weijun Wang, Pingqiang Cai

**Affiliations:** ^1^ Division of Sports Medicine and Adult Reconstructive Surgery, Department of Orthopedic Surgery, Nanjing Drum Tower Hospital, Affiliated Hospital of Medical School Nanjing University Nanjing Jiangsu P. R. China; ^2^ Branch of National Clinical Research Center For Orthopedics Sports Medicine and Rehabilitation P. R. China; ^3^ Co‐innovation Center of Neuroregeneration Nantong University Nantong P. R. China; ^4^ Chemistry and Biomedicine Innovation Center Nanjing University Nanjing P. R. China

**Keywords:** bone–skin crosstalk, diabetic wound, microRNA, neurovascular coupling, tissue clearing

## Abstract

The clinical intractability of diabetic foot ulcers stems from a profound uncoupling of cutaneous neurovascular networks, rendering standard metabolic and topical interventions largely palliative. Paradoxically, remote orthopedic trauma robustly accelerates distal skin repair, yet the systemic molecular mediators driving this “bone‐skin crosstalk” remain undefined, precluding its translation into non‐invasive therapies. Here, we establish that macroscopic bone fracture expedites diabetic wound healing through the systemic release of exosomal miR‐130b‐3p, a potent orchestrator of coupled angiogenesis and neurogenesis. To recapitulate this physiological axis non‐invasively, we engineered a self‐assembling, cholesterol‐modified agomir‐130b‐3p nanocomplex that could bypass endolysosomal degradation. For sustained spatial delivery, these carrier‐free nanoassemblies were incorporated into an in situ photocrosslinkable methacrylated collagen/silk fibroin hydrogel, creating a bio‐instructive extracellular matrix that prolongs microRNA bioavailability. In streptozotocin‐induced diabetic mice, hydrogel‐mediated agomir delivery achieved 97.2% full‐thickness wound closure. Advanced volumetric light‐sheet imaging of chemically cleared whole‐mount skin confirmed the robust spatiotemporal reconstruction of deep vascular and neural networks. These findings decode a distinct exosome‐mediated inter‐organ repair mechanism and demonstrate that biomimetic microRNA nanoformulations can effectively translate systemic physiological cues into localized, high‐efficacy therapeutics for ischemic neuropathic wounds.

## Graphical abstract

This study unravels that bone fracture accelerates diabetic wound healing by releasing exosomal microRNA (miR‐130b‐3p). To mimic this bone‐skin crosstalk, we engineered a self‐assembling agomir‐130b‐3p nanocomplex. Delivered via a photocrosslinkable hydrogel, this bio‐inspired therapy provides sustained localized agomir release. The treatment successfully orchestrates blood vessel and nerve growth, offering an effective, non‐invasive strategy for repairing chronic diabetic ulcers.

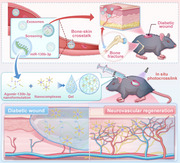

## 1 Introduction

Diabetic foot ulcers (DFUs) are among the most challenging chronic wounds in patients with diabetes mellitus [1]. Current clinical management of DFUs relies on standard‐of‐care interventions, including glycemic control [2], surgical debridement [3], and appropriate dressings [4, 5, 6]. Meanwhile, the field has seen the emergence of therapeutic nanoformulations specifically engineered to target distinct pathological barriers [7, 8, 9], such as multidrug‐resistant infections [10], reactive oxidative stress [11], and impaired skin electro‐conductivity [12, 13]. Despite these multimodal efforts, approximately 25% of DFUs remain unhealed after one year, underscoring the urgent necessity for novel therapeutic strategies [14]. More recently, clinical and preclinical studies have highlighted a fascinating link between skeletal injury and skin repair [15, 16]. Orthopedic procedures such as tibial transverse transport are observed to accelerate the healing of refractory diabetic ulcers in the distal limb, suggesting a systemic bone‐skin crosstalk [17, 18]. Despite their effectiveness, bone remodeling‐based strategies remain invasive procedures with potential risks, including tibial fracture, pin‐tract infection, and anterior tibial skin necrosis [19], highlighting the need to elucidate the underlying molecular mediators of such “bone‐skin crosstalk” to guide the development of mechanistically inspired nanomedicines capable of translating physiological discoveries, such as inter‐organ crosstalk, into stable clinical therapeutics.

Notably, DFUs often involve intertwined neuropathic and ischemic components, implying that coupled nerve and blood vessel impairment is central to their pathogenesis [20]. In diabetic wounds, this neurovascular coupling is severely disrupted; ischemia deprives regenerating nerves of metabolic support, while denervation impairs the secretion of neurotrophic factors essential for vascular stabilization [21, 22, 23]. This vicious cycle of neurovascular dysfunction is a major barrier to healing. Intriguingly, bone remodeling‐based interventions have been associated with improved limb perfusion and peripheral nerve function in clinical studies, which may contribute to accelerated DFU healing [24, 25]. This “bone‐skin crosstalk” aligns with the emerging view of bone as an endocrine organ, capable of releasing extracellular vesicles and soluble factors that modulate homeostasis in extra‐skeletal tissues [26, 27, 28]. However, the specific bone‐derived factor that predominantly modulates neurovascular remodeling in DFUs remains unidentified, representing a critical gap in current knowledge. Furthermore, the clinical application of therapeutic bone fracture is limited by its invasive nature and associated risks, including infection and necrosis. Deciphering the molecular mediator of this bone‐skin axis offers a pathway to develop nanoformulations for non‐invasive therapeutics that recapitulate the benefits of bone remodeling without surgical trauma.

**FIGURE 1:**
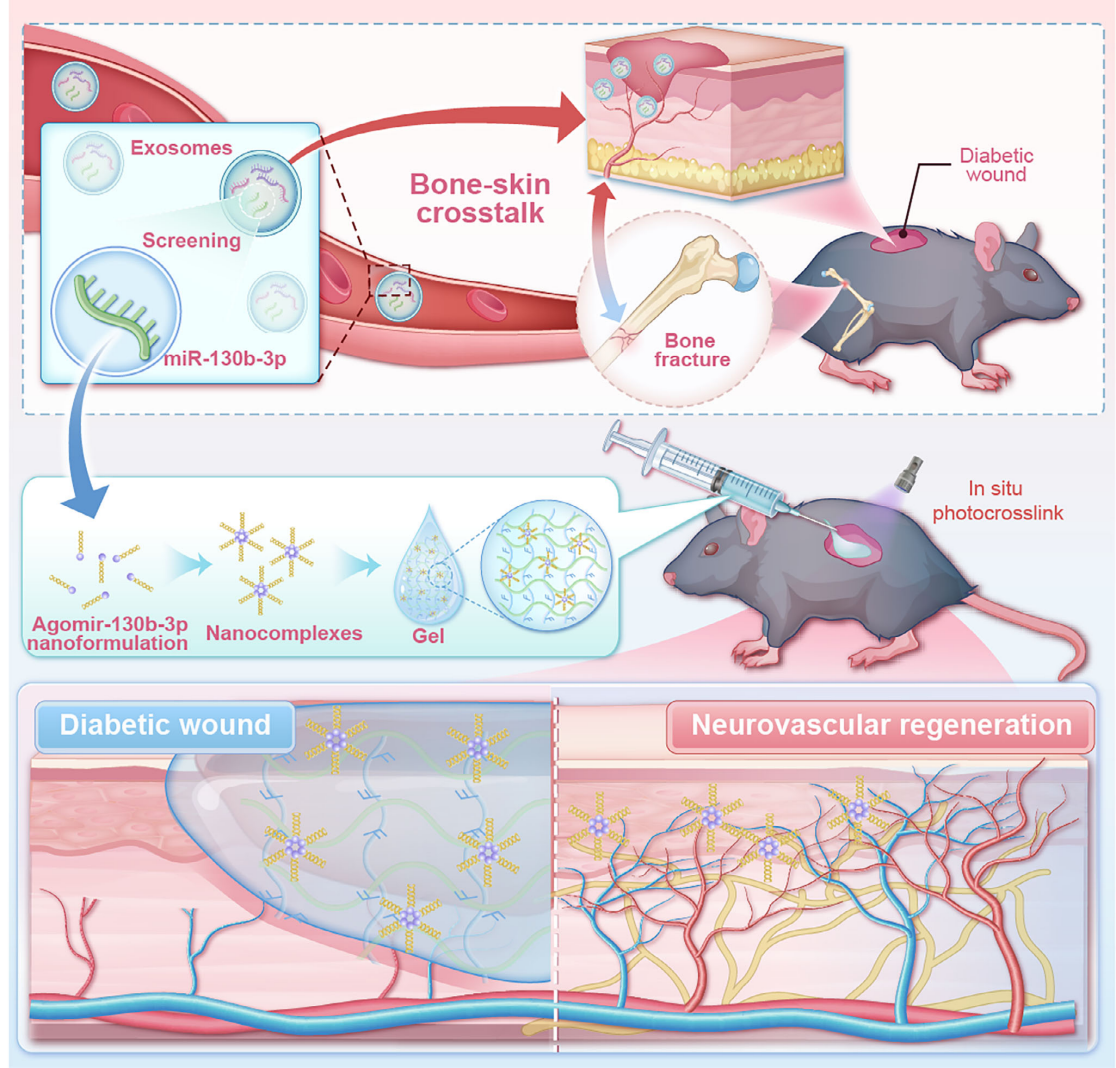
Schematic of the bone‐skin crosstalk‐inspired miRNA nanoformulation for neurovascular regeneration in diabetic wounds. This study leverages a physiological signaling axis in which bone fracture stimulates the release of circulating exosomes enriched with miR‐130b‐3p. These exosomes travel to cutaneous wound sites to orchestrate neurovascular regeneration. To mimic this effect non‐invasively, a synthetic agomir‐130b‐3p nanoformation is chemically modified with cholesterol to form self‐assembling nanocomplexes. These nanocomplexes are encapsulated within an in situ photocrosslinkable collagen/silk fibroin hydrogel (Gel). Upon topical application to a diabetic wound, the hydrogel provides sustained agomir release, promoting coupled angiogenesis and neurogenesis to accelerate full‐thickness healing.

In this study, we identify a bone‐derived exosomal microRNA, miR‐130b‐3p, as a potent mediator of fracture‐induced neurovascular regeneration, thereby promoting diabetic wound repair (Figure 1). Nonetheless, the clinical translation of miRNA therapeutics is hindered by their rapid degradation in biological fluids and poor intracellular uptake. To overcome these barriers, we developed a chemically stabilized agomir‐130b‐3p that spontaneously self‐assembles into cholesterol‐core nanocomplexes. To enable sustained bioavailability and a supportive matrix for cell growth at the wound site, these nanocomplexes were encapsulated in an in situ photocrosslinkable methacrylated collagen/silk fibroin hydrogel. Using whole‐mount tissue clearing and volumetric light‐sheet microscopy, we show that this bio‐inspired nanoformulation orchestrates the 3D reconstruction of the neurovascular network, establishing a carrier‐free, agomir‐based strategy for chronic wound repair.

## 2 Results and Discussion

### 2.1 Bone Fracture Accelerates Diabetic Wound Healing via Circulating Exosomes

**FIGURE 2:**
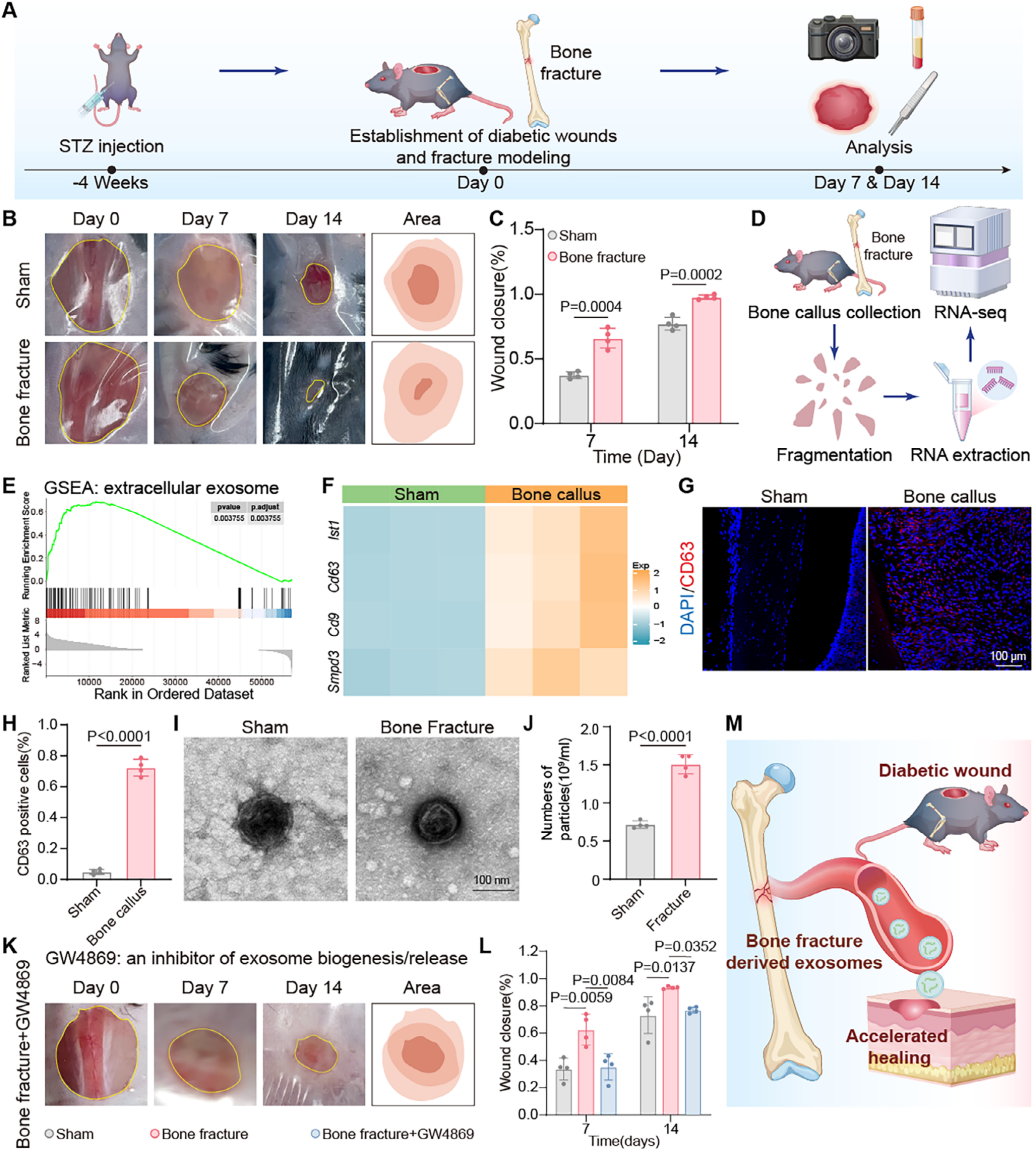
Bone fracture accelerates diabetic wound healing via a systemic exosome‐mediated pathway. (A) Experimental timeline: STZ‐induced diabetic mice received a dorsal full‐thickness excisional wound with or without concomitant femoral fracture, followed by tissue collection on day 7 and 14. (B) Representative photographs of wounds and corresponding wound area traces. All photographs are presented in 1 cm × 1 cm frames. (C) Quantification of wound closure. Data are mean ± SD; *n* = 4 mice per group. (D) Schematic illustration of the experimental workflow for RNA sequencing of fracture callus tissue. (E) Gene set enrichment analysis (GSEA) of the exosome‐associated pathway (GO:0070062). (F) Heatmap showing the expression profiles of representative exosome‐related genes (*Ist1*, *Cd63*, *Cd9*, and *Smpd3*) in fracture callus. (G) Immunofluorescence staining of exosome marker CD63 (red) in fracture and sham tissues; nuclei were counterstained with DAPI (blue). Scale bar, 100 µm. (H) Quantification of CD63‐positive cells. Data are mean ± SD; *n* = 4 mice per group. (I) Transmission electron microscopy (TEM) images of serum‐derived exosomes. Scale bar, 100 nm. (J) Quantification of serum‐derived exosome concentration. Data are mean ± SD. *n* = 4 mice per group. (K) Representative photographs of wounds and corresponding wound area traces. All photographs are presented in 1 cm × 1 cm frames. (L) Quantification of wound closure. Data are mean ± SD; *n* = 4 mice per group. (M) Schematic summary illustrating the association between fracture‐induced exosome release and enhanced wound repair in diabetic mice.

To assess whether bone fracture modulates diabetic wound repair, type I diabetes was induced in mice by streptozotocin (STZ) administration, followed by creation of a full‐thickness excisional dorsal wound in combination with a concurrent femoral fracture (Figure 2A). Compared with non‐fractured controls, fractured diabetic mice exhibited a pronounced acceleration of wound closure, with the wound closure rate increasing from 37.5 ± 2.6% to 66.0 ± 7.4% on day 7, and from 77.3 ± 5.0% to 97.8 ± 1.7% on day 14, respectively. (Figure 2B,C). Histological examination further revealed markedly enhanced re‐epithelialization and collagen deposition in fracture‐associated wounds, as demonstrated by hematoxylin and eosin (H&E) and Masson's trichrome staining (Figure S1A,C). Quantitative analysis of Masson's staining showed that collagen deposition in fractured mice was increased by approximately 1.6‐fold on day 7 and 1.7‐fold on day 14 compared with non‐fractured controls (Figure S1B). Collectively, these findings indicate that bone fracture is associated with enhanced neurovascular remodeling and accelerated wound healing in diabetic mice (Figure S1D).

To explore the molecular basis underlying this phenomenon, RNA sequencing was performed on fracture callus tissue (Figure 2D). Gene Ontology enrichment analysis revealed significant enrichment of biological processes associated with extracellular matrix remodeling, osteogenic activity, and angiogenesis (Figure S2). Given the recognized role of exosomes in mediating inter‐organ communication [29, 30], gene set enrichment analysis (GSEA) was subsequently applied to the exosome‐associated pathway (GO:0070062), which demonstrated robust enrichment of exosome biogenesis‐related signatures (Figure 2E). Multiple core genes involved in exosome formation and secretion, including *Ist1*, *Cd63*, *Cd9*, and *Smpd3*, were also markedly upregulated (Figure 2F). Consistently, immunofluorescence staining confirmed elevated CD63 expression at the fracture site, indicative of enhanced exosome production (Figure 2G,H). Given previous reports suggesting that osteoblasts are a major source of bone‐derived pro‐regenerative exosomes during bone repair [26], we further examined the spatial relationship between exosomal signals and osteoblast‐lineage cells. Co‐immunostaining demonstrated substantial colocalization of CD63‐positive signals with OCN‐positive cells within the fracture callus (Figure S3), supporting the notion that osteoblast‐lineage cells may contribute substantially to the production of fracture‐derived exosomes. Circulating exosomes isolated from fractured mice were validated by transmission electron microscopy (Figure 2I) and western blot analysis of canonical exosomal markers (CD9, CD63, CD81, and TSG101) (Figure S4A). Furthermore, nanoparticle tracking analysis (NTA) revealed a significant increase in serum exosome abundance following fracture (Figure S4B and Figure 2J). To assess the in vivo distribution and wound‐targeting capability of exosomes derived from bone callus, exosomes were first isolated from fracture callus tissue and characterized by transmission electron microscopy (TEM) (Figure S5A), Western blotting for exosomal markers (Figure S5B), and nanoparticle tracking analysis (Figure S5C) to confirm their identity and purity. Subsequently, the well‐characterized exosomes were labeled with DiD and tracked using fluorescence imaging in diabetic mice with full‐thickness dorsal skin wounds. As shown in Figure S5D, fluorescence signals at the wound region progressively increased over time in the exosome‐treated group compared with the PBS‐injected control group. Quantitative analysis further confirmed a time‐dependent accumulation of exosomes at the wound site, indicating their systemic biodistribution and preferential enrichment within the injured tissue within 24 h (Figure S5E). Together, these data suggest that fracture‐induced exosome release might contribute to the augmented reparative capacity observed in diabetic wounds.

To directly test the functional requirement of exosome release, we locally applied GW4869, an inhibitor of exosome biogenesis, to the fracture site. GW4869 markedly reduced exosome production, as shown by decreased CD63 staining at the fracture site (Figure S4C). To exclude the possibility that locally administered GW4869 exerts a systemic effect on exosome secretion in distal skin tissues, we further evaluated exosome marker expression in dorsal skin following local administration of GW4869 near normal bone (without fracture induction). As shown in Figure S6, immunofluorescence analysis of CD63 expression in dorsal skin revealed no significant difference between the GW4869‐treated group and the Sham group (*p* = 0.8410). These results indicate that local GW4869 administration does not directly affect basal exosome‐related signaling in distal skin tissue under physiological conditions. Importantly, inhibition of exosome release abolished the fracture‐induced acceleration of diabetic wound healing, with the wound closure rate decreasing to 35.3 ± 9.4% on day 7 and 76.5 ± 2.1% on day 14, compared with 62.6 ± 10.1% and 93.4 ± 0.9% in untreated fractured mice, respectively (Figure 2K,L and Figure S7A). H&E staining revealed that GW4869 reversed fracture‐enhanced re‐epithelialization (Figure S7B). In parallel, Masson's trichrome staining revealed a marked reduction in collagen deposition following GW4869 treatment (Figure S7C). Quantitative analysis of Masson's staining showed that GW4869 treatment reduced collagen deposition by approximately 1.6‐fold on day 7 and 1.8‐fold on day 14 compared with fractured mice without GW4869 (Figure S7D). Together, these results suggest that exosome release following bone fracture is essential for facilitating angiogenesis, neuroregeneration, and wound closure in diabetic wounds (Figure 2M).

### 2.2 miR‐130b‐3p‐SOCS5 Axis Mediates the Neurovascular Regenerative Effects of Fracture‐Induced Exosomes

**FIGURE 3:**
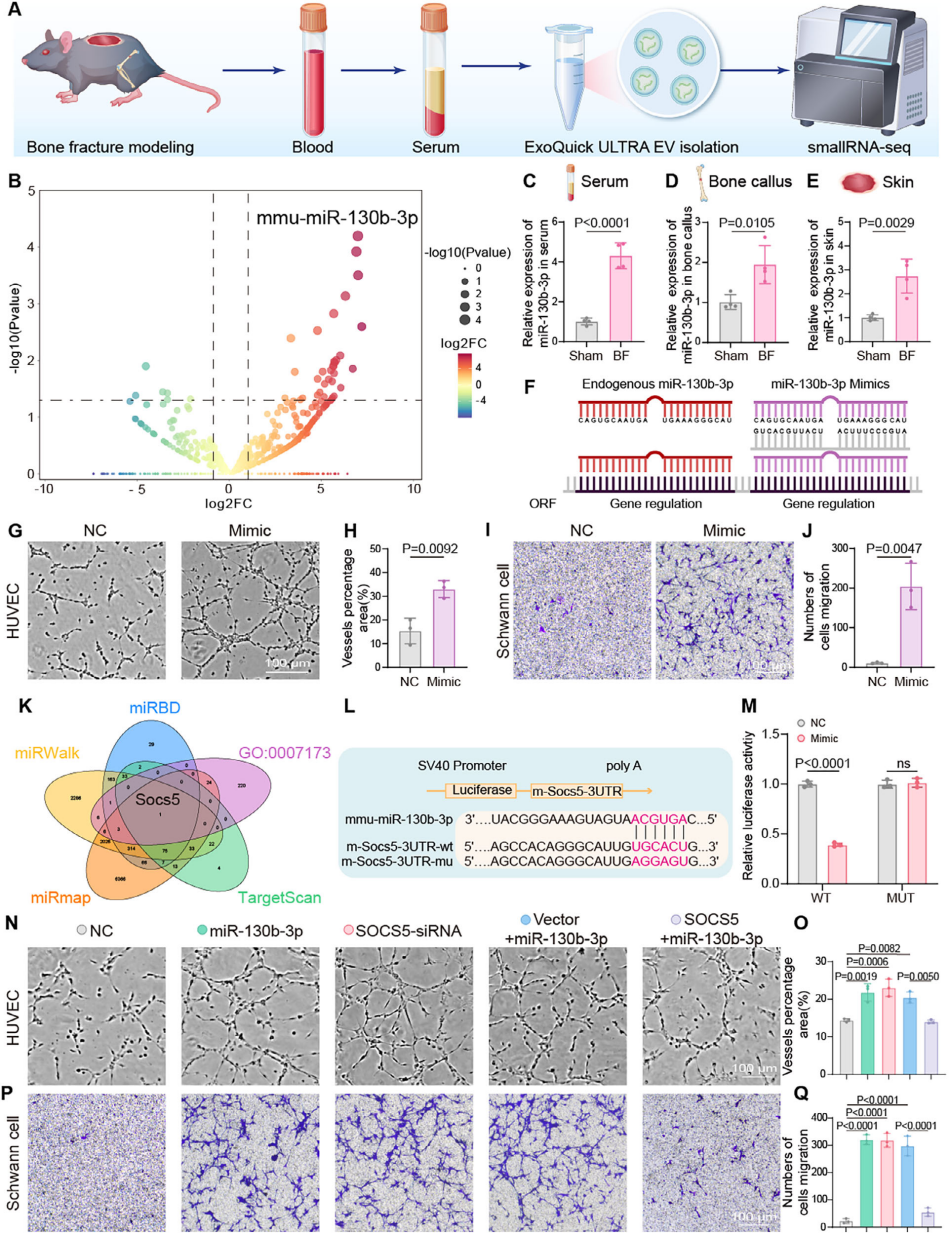
Fracture‐induced miR‐130b‐3p drives exosome–mediated neurovascular coupling by targeting SOCS5. (A) Schematic workflow for small RNA sequencing of serum exosomes isolated from sham and femoral fracture mice (BF). (B) Volcano plot of exosomal small RNA profiles highlighting miR‐130b‐3p. (C–E) RT‐qPCR validation of miR‐130b‐3p levels in serum (C), fracture callus (D), and skin tissue (E). Data are mean ± SD. n = 3 independent biological replicates. (F) Schematic of the synthetic miR‐130b‐3p mimic for gain‐of‐function studies. (G, H) Representative tube formation assays of HUVECs transfected with negative control (NC) or miR‐130b‐3p mimic. Data are mean ± SD; *n* = 3 independent replicates. Scale bars, 100 µm. (I, J) Representative transwell migration assays of Schwann cells (SC) transfected with NC or miR‐130b‐3p mimic. Data are mean ± SD; *n* = 3 independent replicates. Scale bars, 100 µm. (K) Venn diagram showing the overlap between predicted miR‐130b‐3p target genes and genes enriched in the epidermal growth factor signaling pathway (GO: 0007173). (L) Schematic illustration of the dual‐luciferase reporter assay. (M) Dual‐luciferase reporter assays confirming the direct interaction between miR‐130b‐3p and the 3′ untranslated region (3′UTR) of SOCS5. Data are mean ± SD. *n* = 3 independent biological replicates. (N) Functional experiments in HUVECs examining the effects of SOCS5 overexpression on miR‐130b‐3p–regulated tube formation. Scale bar, 100 µm. (O) Quantitative analysis of the vessels percentage area(%) in HUVECs. Data are mean ± SD. n = 3 independent biological replicates. (P) Functional experiments in Schwann cells examining the effects of SOCS5 overexpression on miR‐130b‐3p–regulated cell migration. Scale bar, 100 µm. (Q) Quantitative analysis of the number of SCs migration. Data are mean ± SD. *n* = 3 independent biological replicates.

To identify potential molecular mediators underlying the pro‐healing activity of fracture‐derived exosomes, serum exosomes were isolated from mice following femoral fracture and subjected to small RNA sequencing (Figure 3A). Pathway enrichment analyses highlighted significant activation of actin cytoskeleton remodeling, cell growth, and axon development pathways, suggesting a potential role for exosomal small RNAs in enhancing angiogenesis and neuroregeneration (Figure S8). Volcano plot analysis identified miR‐130b‐3p as the most prominently upregulated small RNA (Figure 3B). Consistently, miR‐130b‐3p levels were markedly elevated following fracture, exhibiting an approximately 4.1‐fold increase in serum (Figure 3C), a 2.1‐fold increase in fracture callus (Figure 3D), and a 2.8‐fold increase in skin tissue (Figure 3E), compared with non‐fractured controls. However, the role of miR‐130b‐3p in neurovascular regeneration had not been previously established.

A miRNA mimic is a synthetic RNA molecule that recapitulates the function of endogenous small RNAs and serves as a tool for gain‐of‐function studies (Figure 3F) [31, 32]. To assess functional relevance, a miR‐130b‐3p mimic was transfected into human umbilical vein endothelial cells (HUVECs) and Schwann cells (SCs), resulting in robust intracellular elevation of miR‐130b‐3p (Figure S9A,B). Cell Counting Kit‐8 (CCK‐8) assays indicated that the mimic had little effect on cell proliferation (Figure S9C,D). In contrast, tube formation assays showed that miR‐130b‐3p substantially enhanced angiogenic capacity in HUVECs, resulting in an approximately 2.2‐fold increase in vascular area compared with controls (Figure 3G,H), while Transwell assays revealed significant promotion of SCs migration, with the number of migrated cells increasing by approximately 3.5‐fold following miR‐130b‐3p treatment (Figure 3I,J). These data indicate that miR‐130b‐3p functions as a regenerative small RNA that enhances angiogenesis and nerve regeneration. To further investigate whether miR‐130b‐3p promotes functional neurovascular coupling, a Transwell co‐culture system consisting of HUVECs and SCs was established. SCs transfected with miR‐130b‐3p significantly enhanced the angiogenic activity of co‐cultured HUVECs, resulting in an approximately 1.3‐fold increase in vascular area compared with the NC group (Figure S10A,B). Conversely, miR‐130b‐3p‐transfected HUVECs markedly promoted SC migration, with the number of migrated cells increasing by approximately 4.9‐fold (Figure S10E,F). Furthermore, ELISA analysis demonstrated that miR‐130b‐3p enhanced the secretion of angiogenic factors by SCs, including a 2.1‐fold increase in VEGF and a 1.7‐fold increase in PDGF‐BB (Figure S10C,D). In parallel, HUVECs transfected with miR‐130b‐3p exhibited elevated secretion of neurotrophic factors, with NGF and BDNF levels increasing by approximately 2.5‐fold and 1.8‐fold, respectively (Figure S10G,H). These findings suggest that miR‐130b‐3p facilitates bidirectional endothelial‐Schwann cell communication by promoting the reciprocal release of angiogenic and neurotrophic mediators, thereby contributing to coordinated neurovascular regeneration. These findings pointed to miR‐130b‐3p as a central mediator, so we next investigated its molecular target and therapeutic potential. To recognize downstream targets, candidate genes were predicted using miRDB, miRWalk, miRmap, and TargetScan, and intersected with genes enriched in the epidermal growth factor signaling pathway (GO: 0007173). Suppressor of cytokine signaling 5 (*Socs5*) was identified as a potential target (Figure 3K). Dual‐luciferase reporter assays confirmed a direct interaction between miR‐130b‐3p and the *Socs5* 3′UTR (Figure 3L,M). qPCR and Western blot analyses further demonstrated that miR‐130b‐3p mimic markedly reduced *Socs5* mRNA and protein levels in HUVECs (Figure S11A,B) and SCs (Figure S11C,D). To assess the functional relevance of the miR‐130b‐3p‐SOCS5 axis, SOCS5 was overexpressed in HUVECs and SCs. Overexpression of SOCS5 abolished the miR‐130b‐3p‐induced enhancement of tube formation in HUVECs (Figure 3N,O) and of SC migration (Figure 3P,Q), demonstrating that this pathway regulates both angiogenic and neuroregenerative responses. Western blot analysis confirmed the effects of miR‐130b‐3p mimic and SOCS5 plasmid on SOCS5 protein levels in these cells (Figure S12A,B). In addition, the effects of miR‐130b‐3p were also evaluated in human dermal fibroblasts (HDFs), with consistent pro‐migratory and SOCS5‐dependent responses observed (Figure S13 and S14). Together, these findings establish the miR‐130b‐3p‐SOCS5 axis as a key molecular mechanism mediating the pro‐angiogenic and pro‐regenerative effects of fracture‐derived exosomes.

### 2.3 Synthetic Agomir‐130b‐3p Nanocomplexes Enable Efficient Cellular Uptake

**FIGURE 4:**
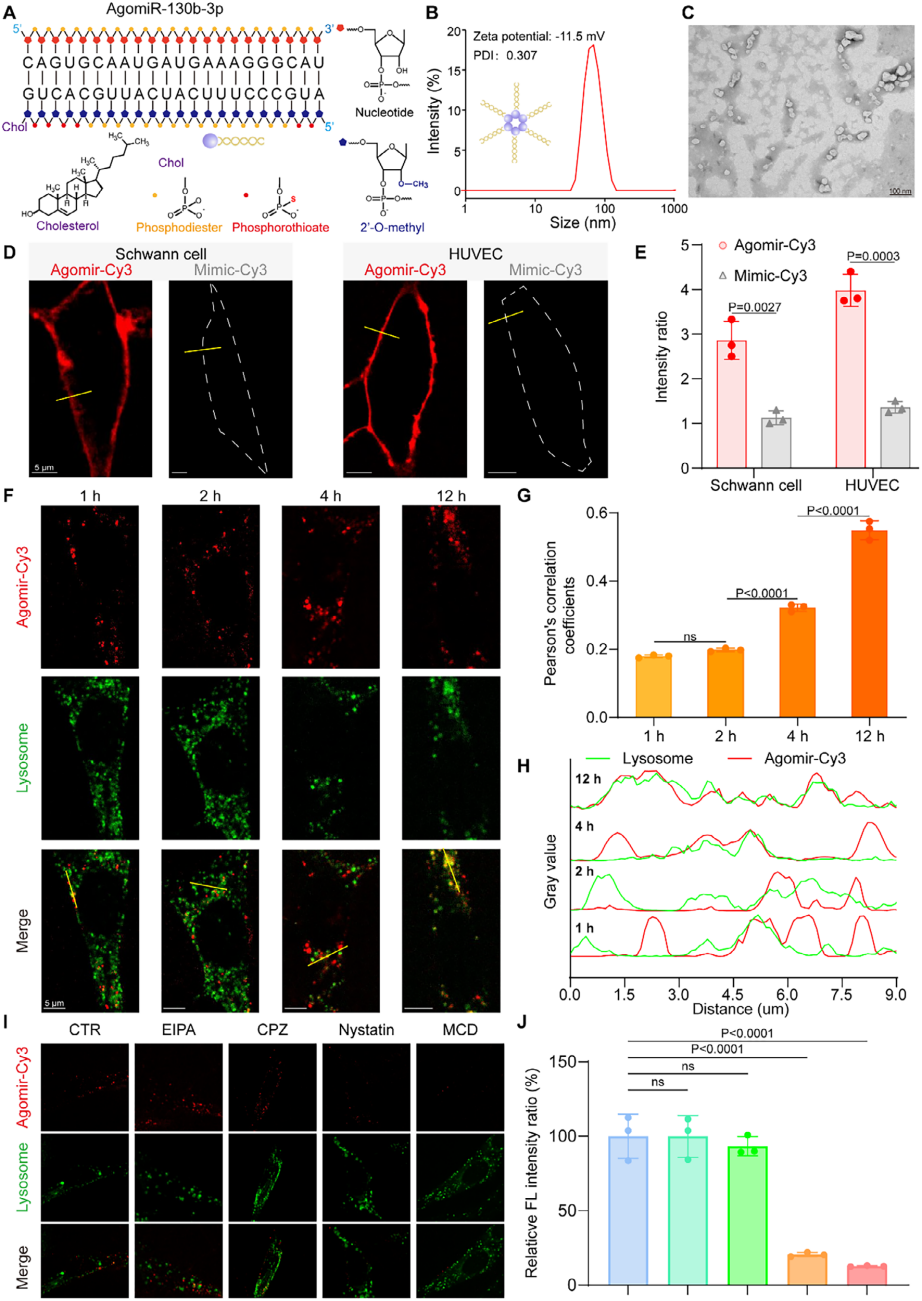
Engineering and cellular uptake of self‐assembling cholesterol‐modified agomir‐130b‐3p nanocomplexes. (A) Chemical structure and sequence of agomir‐130b‐3p, incorporating terminal phosphorothioate (PS) linkages, 2'‐O‐methyl (2'‐OMe) modifications, and a 3'‐terminal cholesterol conjugate. (B) Hydrodynamic diameter distribution of agomir nanoassemblies measured by dynamic light scattering (DLS). (C) Representative transmission electron microscopy (TEM) image of the agomir nanoassemblies. (D) Confocal imaging of live Schwann cells and HUVEC incubated with Cy3‐labeled agomir (agomir‐cy3) or an unmodified Cy3‐labeled mimic (mimic‐cy3) at 5 min, dashed line denotes the cytomembrane. It shows high affinity of agomir nanocomplexes to cytomembranes of Schwann and HUVEC cells. (E) Ratio of fluorescence intensity at the cytomembrane to the background, the yellow line of (D) indicates the region used for intensity quantification. (F) Time‐lapse confocal imaging of a live Schwann cell incubated with agomir‐Cy3 (red) and Lysosome trackers (green) within 12 h. It shows agomir nanocomplexes can be efficiently internalized within 1 h and remain within the cell for 12 h. (G) Pearson correlation coefficients of agomir‐Cy3 (red) and Lysosome (green). It shows limited co‐localized signals of the internalized agomir‐Cy3 with lysosome markers. (H) Line profile of fluorescence intensity within the Schwann cell. The profile was measured along the line indicated in yellow in the inset of (F). (I) Confocal images of Agomir‐Cy3 (red) and Lysotracker (green) in SCs treated with CTR, EIPA, CPZ, Nystatin, or MCD. (J) Quantitative analysis of the relative fluorescence intensity of Agomir‐Cy3 in SCs under the indicated treatments. Data are mean ± SD. *n* = 3 independent biological replicates. ns, not significant.

To enable the stability and cellular uptake of miR‐130b‐3p, we developed a chemically modified agomir‐130b‐3p. This construct is uniformly referred to as agomir throughout this study. The synthetic agomir is an engineered double‐stranded RNA molecule designed to mimic the mature miRNA for gain‐of‐function studies. Its antisense strand is systematically modified to: it features two phosphorothioate linkages at the 5′ terminus, four phosphorothioate linkages at the 3′ terminus, a 3′‐terminal cholesterol moiety, and comprehensive 2′‐methoxy (2′‐OMe) modifications across all nucleotides (Figure 4A). RNase A treatment showed that agomirs have stronger resistance to nuclease degradation than unmodified mimics (Figure S15). Furthermore, we found that cholesterol‐modified agomirs spontaneously self‐assembled into nanoassemblies in aqueous solution. Dynamic light scattering (DLS) analysis indicated that the nanoassemblies had a hydrodynamic diameter of approximately 80 nm, with a polydispersity index (PDI) of 0.307 and a zeta potential of −11.5 mV (Figure 4B and Figure S16), Transmission electron microscopy (TEM) imaging revealed a diameter of 30–50 nm (Figure 4C). Such architecture arises from molecular self‐assembly, where hydrophobic cholesterol moieties overcome electrostatic repulsion between agomir strands to drive core formation, resulting in stable nanoassembled structures [33, 34, 35].

Subsequently, we explored the cellular uptake dynamics of agomirs compared to unmodified mimics. Spinning‐disk confocal microscopy imaging showed the uptake of agomir‐Cy3 by live SCs and HUVECs. Notably, the agomir‐Cy3 exhibited excellent plasma membrane affinity, enriching on the membrane surface within 5 min due to the modification of cholesterol; no such localization was observed with the unmodified Mimic‐Cy3 (Figure 4D). Quantitative analysis of the fluorescence Ratio intensity at the cytomembrane to the background further corroborated these observations; the fluorescence intensity at the cell membrane was 2.9 to 4.0 times higher than the background (Figure 4E).

To distinguish whether agomir was transported to lysosomes after internalization, the cells were stained with a lysosome tracker. Agomirs exhibited a progressive, time‐dependent intracellular accumulation over 12 h. However, fluorescence microscopy analysis revealed the limited colocalization of Agomir‐Cy3 with lysosomal markers; the Pearson correlation coefficient increased from 0.18 at 1 h to 0.55 at 12 h, suggesting that a portion might partially achieve endosomal escape (Figure 4F–H). To identify the cellular entry pathway of the cholesterol‐modified agomir nanoparticles, we treated SCs with a panel of pharmacological inhibitors targeting distinct endocytic routes (Figure 4I,J). Compared with the untreated control (CTR), neither EIPA (macropinocytosis inhibitor) nor CPZ (clathrin‐mediated endocytosis inhibitor) significantly reduced the cellular uptake of Agomir‐Cy3, as reflected by the unchanged relative fluorescence intensity. In contrast, the lipid raft/caveolae‐mediated endocytosis inhibitors Nystatin and MCD drastically reduced Agomir‐Cy3 internalization, with only 20.5 ± 1.4% and 12.8 ± 0.4% of uptake remaining, respectively. These results indicate that the nanoparticles are primarily taken up by cells through the lipid raft/caveolae‐dependent pathway, rather than via clathrin‐mediated endocytosis or macropinocytosis. We demonstrated the effective delivery of agomir in SCs and HUVECs, laying the foundation for subsequent therapeutic applications.

### 2.4 Photocrosslinkable Composite Hydrogel Prolongs Agomir‐130b‐3p Retention

To enable sustained agomir bioavailability, we designed a methacrylated collagen silk fibroin (ColMA/SF) composite hydrogel. The composite hydrogel also matched the mechanical support required for wound healing and the dynamic degradation process [36]. A novel photocrosslinkable metharylated collagen was first synthesized, through chemical modification of rat tail type I collagen with glycidyl methacrylate (Figure S17A). The introduction of the methacryloyl double bond was confirmed with ^1^H Nuclear Magnetic Resonance spectrum, showing characteristic peaks at chemical shifts of 5.61 and 6.03 ppm (Figure S17B) [37]. This modification strategy led to a reduction in the molecular weight of collagen. Consequently, it enhanced its solubility in phosphate‐buffered saline (PBS) to over 50 mg/mL, thereby facilitating the preparation of high‐concentration precursors (Figure S17C). We used 40 mg/mL ColMA (ColMA_40_) as the foundational hydrogel matrix, based on the gelation time and solubility. The hydrogel can undergo gelation within 2 min under 365 nm UV irradiation, exhibiting excellent in situ wound conformability and mechanical tolerance to twisting (Figure S17D).

To optimize the mechanical and biological performance of the collagen hydrogel, the highly biocompatible silk fibroin was incorporated into the ColMA matrix [38, 39, 40]. First, a preliminary cell proliferation assay was conducted to screen the optimal SF concentration (0–20 mg/mL). Live/dead cell staining assays showed that incorporation of 10 mg/mL SF (SF_10_) produced the strongest proliferative effect, increasing viable cell numbers to approximately 1.5‐fold of control levels in HUVECs and 2.6‐fold in SCs (Figure S18A). This trend was further supported by 5‐Ethynyl‐2′‐deoxyuridine (EdU) analysis. In HUVECs, the proportion of EdU‐positive cells increased from 34.0% in the control group to 43.0% following SF_10_ treatment. Similarly, in SCs, the EdU‐positive fraction rose markedly from 9.8% in controls to 35.6% with SF_10_ incorporation (Figure S18B). Based on these assessments, SF_10_ was selected for subsequent experiments. Observation of the freeze‐dried hydrogel microstructure using scanning electron microscopy revealed that the pure ColMA hydrogel (ColMA_40_) presented heterogeneous pore morphology. In contrast, the incorporation of SF (ColMA_40_SF_10_) resulted in a uniform and dense pore structure (Figure S19A). Cyclic compression tests demonstrated that the ColMA_40_SF_10_ hydrogel exhibited excellent viscoelastic recovery and maintained structural integrity through 10 cycles at 50% strain (Figure S19B). Quantitative data showed that incorporating SF increased the compressive modulus of the hydrogel from 3.5 kPa to 5.4 kPa (Figure S19C). The storage modulus (G′) of the ColMA_40_SF_10_ hydrogel was consistently higher than the loss modulus (G″) across the tested frequency range, indicating the formation of a stable three‐dimensional network structure and typical solid hydrogel behavior. Compared to the pure ColMA hydrogel, the composite hydrogel exhibited higher modulus values and weaker frequency dependence, indicating that the introduction of SF enhanced the crosslinking density and network stability, making it more adaptable to dynamic physiological environments (Figure S19D).

The biological effects of the composite hydrogel were then examined. Live/dead cell staining assays showed that the ColMA_40_SF_10_ hydrogel (Gel) increased viable cell numbers relative to the control, rising from 60.0 to 77.3 in HUVECs and from 81.3 to 185.7 in SCs. Notably, comparable viable cell numbers were observed in the Gel, negative control RNA‐loaded hydrogel group (NC@Gel), and agomir‐loaded hydrogel group (Agomir@Gel) groups (Figure S20A). Consistently, EdU incorporation revealed increased proliferation with Gel treatment, with EdU‐positive fractions increasing from 18.8% to 28.5% in HUVECs and from 8.5% to 20.3% in Schwann cells. At the same time, no significant differences were detected among the three hydrogel‐based groups (Figure S20B). Similarly, HDFs showed comparable cytocompatibility (Figure S21). This strongly demonstrates that the incorporation and sustained release of agomirs exhibit good biocompatibility, do not induce additional cytotoxicity, and ensure treatment safety. Meanwhile, the biodegradability of hydrogels is critical for tissue regeneration. The ColMA_40_SF_10_ hydrogel showed progressive in vitro degradation, as quantified by gravimetric analysis. The mass decreased to 14.9% of the initial dry weight after 7 days, and approached complete disintegration by day 14 (Figure S22A).

Lastly, the agomir release kinetics in the ColMA_40_SF_10_ hydrogel were studied. It showed a sustained‐release profile of agomir, with a cumulative release ratio of 85.2% over 14 days (Figure S22B). Considering the persistent oxidative stress and elevated MMP‐9 expression in diabetic wounds [41], we further investigated the antioxidant activity and in vivo degradation behavior of the hydrogel. The free radical scavenging activities of ColMA_40_ and ColMA_40_SF_10_ were evaluated against DPPH and hydroxyl (·OH) radicals (Figure S23). ColMA_40_SF_10_ exhibited a higher DPPH radical‐scavenging rate (71.0 ± 5.5%) than ColMA_40_ (59.0 ± 8.5%), indicating enhanced antioxidant capacity after SF incorporation. Both hydrogels also showed strong scavenging activity against ·OH radicals, with average scavenging rates of 80.8 ± 1.7% for ColMA_40_ and 84.1 ± 7.2% for ColMA_40_SF_10_. These findings suggest that the introduction of SF further improved the antioxidant performance of the ColMA‐based hydrogel. To evaluate hydrogel stability under pathological wound conditions, the in vivo degradation profile was monitored by real‐time fluorescence imaging over 12 days in a mouse full‐thickness wound model (Figure S24A). Quantitative analysis of the residual fluorescence intensity (Figure S24B) revealed a gradual and continuous degradation process rather than an abrupt breakdown. The fluorescence signal steadily decreased from day 1 to day 12, with near‐complete degradation observed by day 12. Notably, no sudden decline in fluorescence intensity was detected throughout the observation period, indicating that the hydrogel maintained structural integrity during degradation even within the ROS‐ and MMP‐rich wound microenvironment. Collectively, these results suggest that the ColMA_40_SF_10_ hydrogel possesses favorable antioxidant properties and undergoes controlled degradation in diabetic wounds, thereby supporting sustained agomir release throughout the therapeutic period.

### 2.5 Sustained Agomir‐130b‐3p Release Accelerates Wound Healing

**FIGURE 5:**
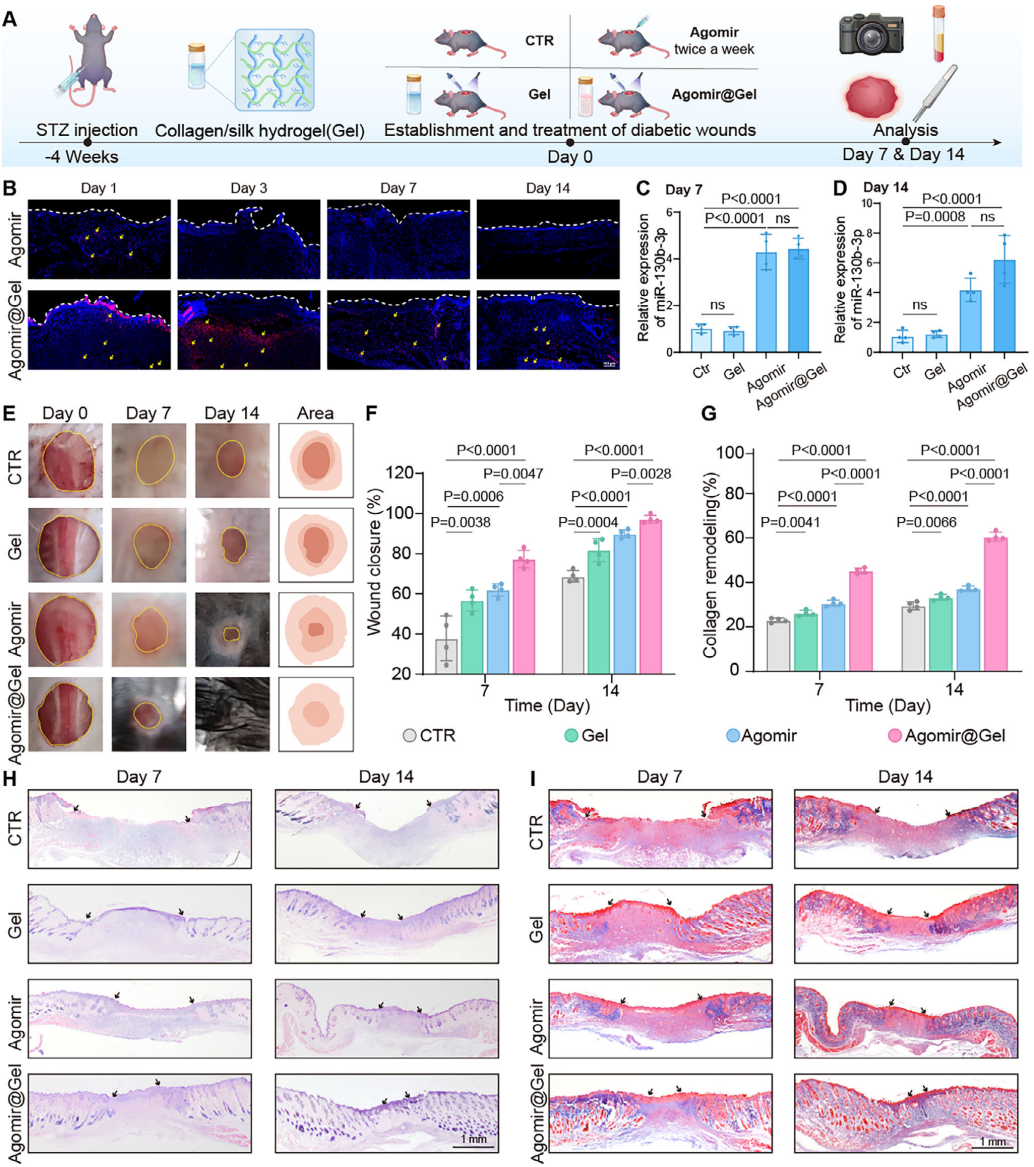
Agomir‐loaded photocrosslinkable hydrogel enables sustained miRNA release and accelerated diabetic wound healing. (A) Experimental timeline: STZ‐induced diabetic mice received a dorsal full‐thickness excisional wound and were treated with vehicle control (CTR), blank hydrogel (Gel), free agomir‐130b‐3p (Agomir), or agomir‐130b‐3p–loaded hydrogel (Agomir@Gel); followed by tissue collection on day 7 and 14. (B) In vivo release profile of agomir from Agomir@Gel compared with Agomir. (C) qPCR analysis of miR‐130b‐3p expression in wound tissues on day 7 after treatment with CTR, Gel, Agomir, or Agomir@Gel. Data are mean ± SD; n = 4 mice per group. (D) qPCR analysis of miR‐130b‐3p expression in wound tissues on day 14 after treatment with CTR, Gel, Agomir, or Agomir@Gel. Data are mean ± SD; n = 4 mice per group. (E) Representative photographs of wounds and corresponding wound area traces. All photographs are presented in 1 cm × 1 cm frames. (F, G) Quantification of wound closure (F) and collagen remodeling (G). Data are mean ± SD; *n* = 4 mice per group. (H, I) Representative H&E (H) and Masson's trichrome (I) staining of wound sections at the indicated time points. Arrows denote the wound margins. Scale bar, 1 mm.

To further evaluate the in vivo sustained delivery of miR‐130b‐3p and the therapeutic potential of Agomir@Gel, STZ‐induced diabetic mice were subjected to a full‐thickness excisional dorsal wound and randomly assigned to four groups: CTR, Gel, Agomir, and Agomir@Gel (Figure 5A). Compared with a single bolus injection of an equal amount of agomir, the hydrogel enabled a gradual and sustained release of agomir (Figure 5B). Accordingly, in subsequent experiments, free agomir was delivered twice weekly, while the agomir‐loaded hydrogel was administered as a single dose to ensure full biological activity of agomir and allow direct comparison between groups. Quantitative qPCR analysis showed that miR‐130b‐3p expression remained at baseline levels in the control group (CTR) and Gel groups during the 14 days. On day 7, agomir treatment markedly increased miR‐130b‐3p expression (∼4.2‐fold compared to CTR), while Agomir@Gel induced a comparable elevation (∼4.4‐fold) with reduced variability (Figure 5C). By day 14, miR‐130b‐3p levels declined in the agomir group (∼3.2‐fold compared to CTR) but remained higher with Agomir@Gel (∼5.9‐fold) (Figure 5D), indicating a prolonged availability of miRNA at the wound site. Through chemical modification and compositing, the platform achieved in‐situ photocurable formation, enhanced mechanical properties and stability, and sustained agomir release. Wound healing capability was subsequently assessed. On day 7, wound closure was limited in the CTR group (37.9 ± 11.4%), moderately improved with Gel (56.8 ± 5.2%) and Agomir alone (62.0 ± 3.0%), and most pronounced in the Agomir@Gel group (77.3 ± 4.5%). On day 14, Agomir@Gel treatment resulted in near‐complete wound closure (97.2 ± 1.9%), indicating the greatest and most consistent therapeutic benefit. By contrast, Gel and Agomir treatments achieved higher closure rates (81.8 ± 5.7% and 89.9 ± 2.0%, respectively), while wounds remained partially unhealed in CTR mice (68.8 ± 2.8%). (Figure 5E,F) Likewise, H&E and Masson's staining showed enhanced re‐epithelialization and collagen deposition across all treatment groups, with the Agomir@Gel outperforming other groups (Figure 5H,I). On day 7, collagen deposition was modestly increased in the Gel (∼1.1‐fold) and Agomir groups (∼1.3‐fold) relative to CTR, whereas the Agomir@Gel group exhibited a nearly 2.0‐fold enhancement. By day 14, this difference became more pronounced: Gel and Agomir treatments achieved ∼1.1‐fold and ∼1.3‐fold increases, respectively, while the Agomir@Gel composite hydrogel sustained a robust ∼2.1‐fold elevation compared with the CTR group (Figure 5G). Collectively, these results demonstrate that the Agomir@Gel maximizes reparative responses in diabetic wounds.

Notably, H&E staining of major organs (heart, liver, spleen, lung, kidney) (Figure S25) and serum levels of alanine aminotransferase (ALT), aspartate aminotransferase (AST), creatinine (CREA), and urea (UREA) (Figure S26) revealed few pathological abnormalities, implying the Agomir@Gel's excellent biocompatibility and absence of systemic toxicity. Collectively, these results demonstrate that the Agomir@Gel maximizes angiogenic, neurodegenerative, and reparative responses in diabetic wounds.

### 2.6 Agomir‐130b‐3p Orchestrates Neurovascular Network Reconstruction

**FIGURE 6:**
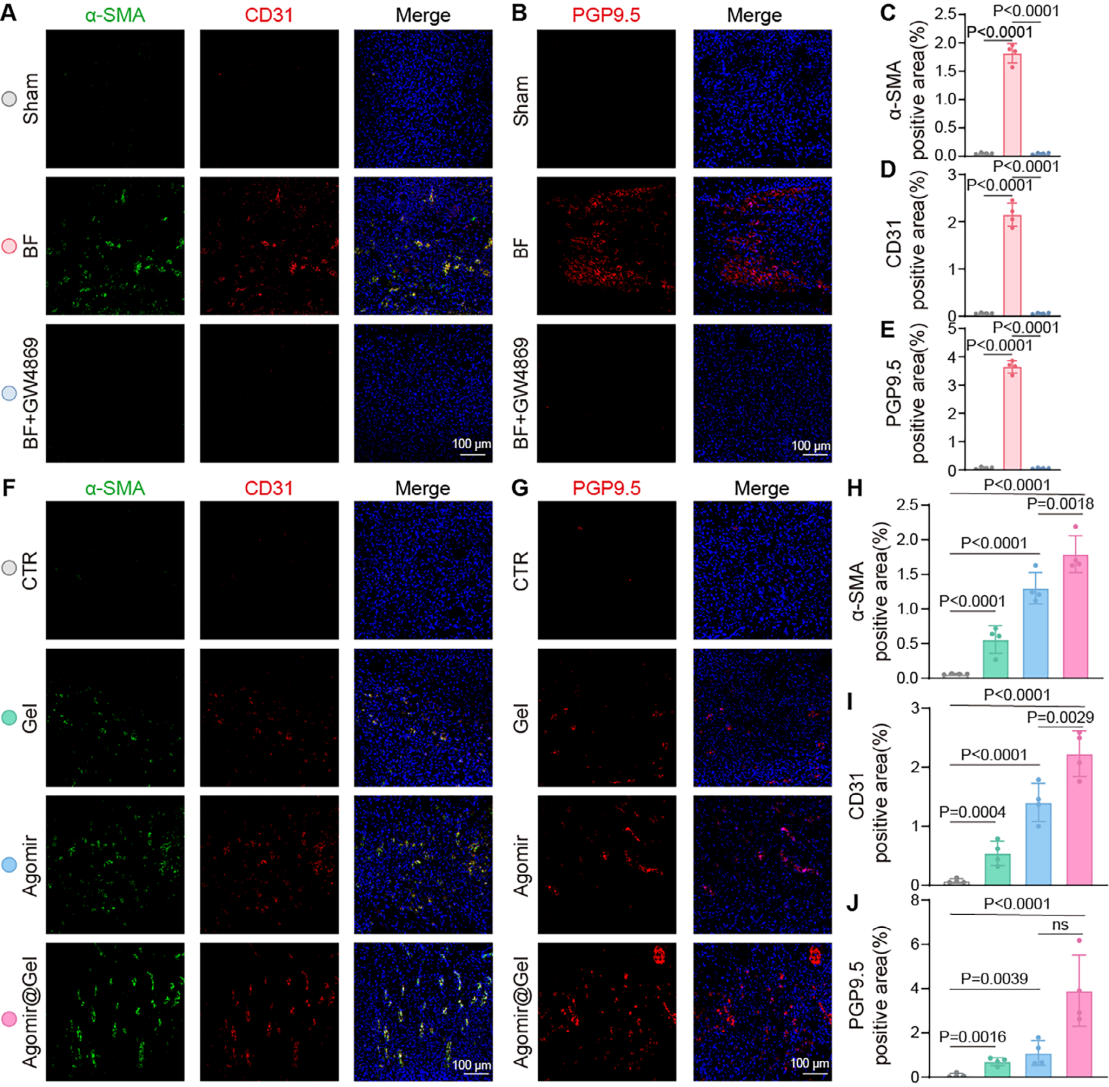
Fracture‐derived exosomes and Agomir@Gel therapy promote robust neurovascular regeneration in vivo. (A) Immunofluorescence staining of α‐smooth muscle actin (α‐SMA, green) and CD31 (red) with DAPI nuclear counterstain (blue) in Sham, BF, and BF+GW4869 groups. Scale bar, 100 µm. (B) Immunofluorescence staining of the pan‐neuronal marker PGP9.5 (red) with DAPI (blue) in the same groups. Scale bar, 100 µm. (C–E) Quantification of α‐SMA (C), CD31 (D), and PGP9.5 (E) positive cells in Sham, BF, and BF+GW4869 groups. Data are mean ± SD; *n* = 4 mice per group. (F) Immunofluorescence staining of α‐SMA (green) and CD31 (red) with DAPI (blue) in CTR, Gel, Agomir, and Agomir@Gel groups. Scale bar, 100 µm. (G) Immunofluorescence staining of PGP9.5 (red) with DAPI (blue) in the same groups. Scale bar, 100 µm. (H–J) Quantification of α‐SMA (H), CD31 (I), and PGP9.5 (J) positive cells in CTR, Gel, Agomir, and Agomir@Gel groups. Data are mean ± SD; *n* = 4 mice per group.

Although the beneficial effects of fracture‐derived exosomes and Agomir@Gel were evident, their roles in neurovascular remodeling require further investigation. Immunofluorescence staining demonstrated that exosomes serve as a critical mediator of bone fracture‐induced neurovascular regeneration. Robust angiogenesis was observed in fractured mice, as evidenced by markedly increased α‐SMA and CD31 staining compared with sham controls (Figure 6A). Quantitative analysis revealed that the α‐SMA‐positive area was approximately 35‐fold higher in the BF group. In contrast, pharmacological inhibition of exosome release with GW4869 almost completely abolished this increase, restoring α‐SMA levels to levels comparable to those in the sham group (Figure 6C). A similar pattern was observed for CD31, with an approximately 34‐fold increase following bone fracture, which was largely eliminated by GW4869 treatment (Figure 6D). Neurogenesis, assessed by PGP9.5 immunostaining, exhibited a parallel trend (Figure 6B). The PGP9.5‐positive area was dramatically increased (49‐fold) in fractured mice relative to sham controls, indicating robust nerve regeneration induced by bone fracture. Notably, suppression of exosome secretion by GW4869 nearly abolished this neurogenic effect, resulting in PGP9.5 levels comparable to baseline (Figure 6E). Building on this pivotal role of fracture‐derived exosomes in mediating neurovascular remodeling, we next evaluated whether Agomir@Gel could further enhance angiogenesis and neurogenesis. Consistently, treatment with Gel, Agomir, or Agomir@Gel resulted in robust neovascularization and nerve regeneration. Immunofluorescence staining demonstrated enhanced angiogenesis, as indicated by increased α‐SMA and CD31 staining (Figure 6F). The α‐SMA‐positive area increased by 9.3‐fold, 21.7‐fold, and 29.8‐fold (Figure 6H), and the CD31‐positive area increased by 7.2‐fold, 18.8‐fold, and 29.7‐fold in the Gel, Agomir, and AgomiR@Gel groups, respectively, relative to controls (Figure 6I). Neurogenesis, assessed by PGP9.5 staining, was also significantly elevated (Figure 6G), with increases of 8.1‐fold, 12.6‐fold, and 44.7‐fold in the Gel, Agomir, and Agomir@Gel groups, respectively (Figure 6J). These results highlight that both Gel and Agomir significantly promoted angiogenesis and neurogenesis, with the combination (Agomir@Gel) eliciting the most robust neovascularization and nerve regeneration.

**FIGURE 7:**
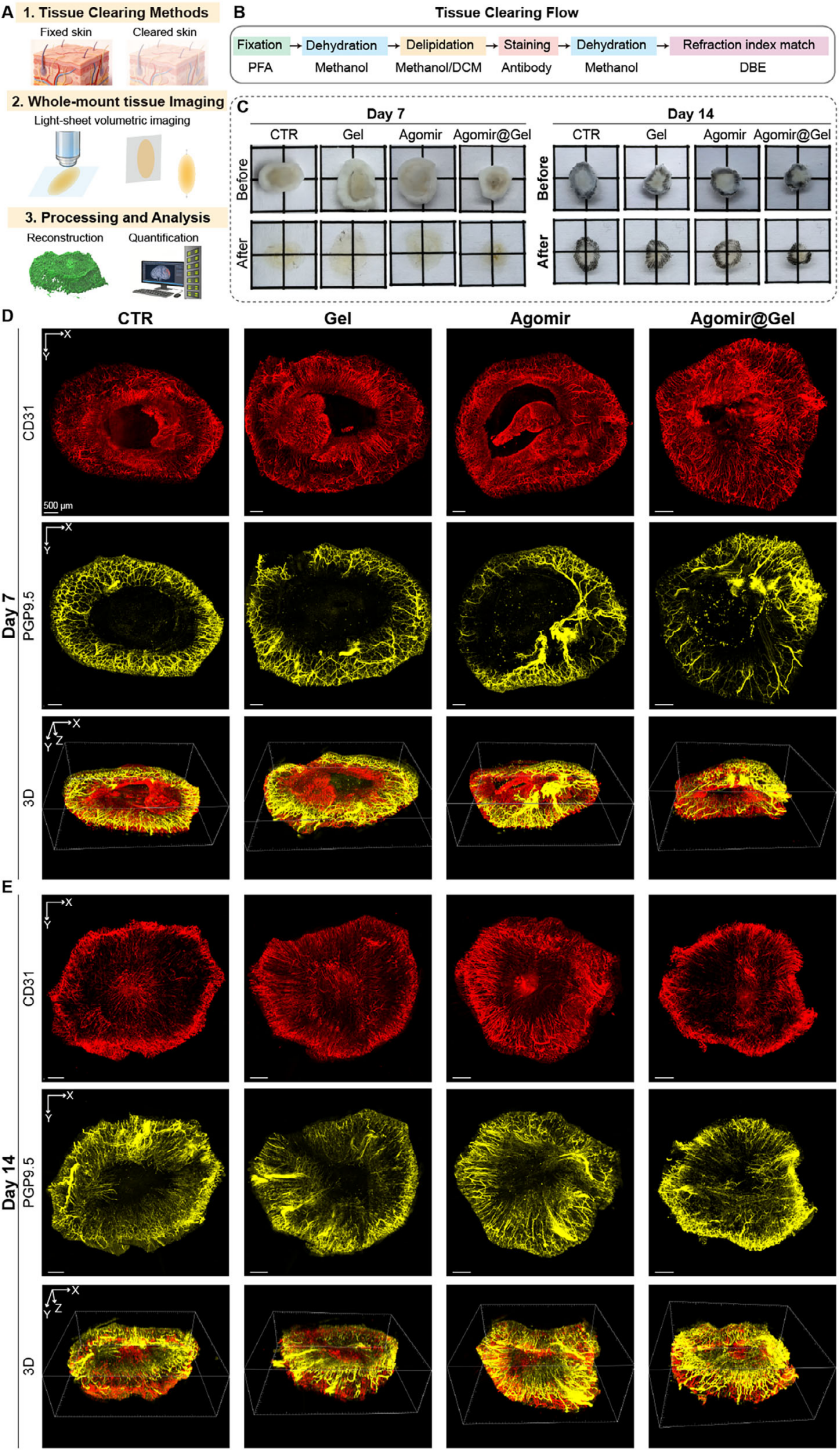
Volumetric light‐sheet imaging of cleared skin reveals the full‐thickness neurovascular networks. (A) Overview of the 3D imaging pipeline, comprising tissue clearing, light‐sheet optical tomography, and computational reconstruction. (B) Schematic Workflow of the iDISCO+ tissue clearing protocol, including fixation, serial dehydration, delipidation, immunostaining, and refractive index matching for optical transparency. (C) Photographs of skin samples before (fixed) and after (cleared) processing, demonstrating high optical transparency. (D, E) Representative 3D maximum intensity projections (upper and middle rows) and 3D view (lower row) of the full‐thickness wound bed stained for CD31 (vasculature, red) and PGP9.5 (nerves, yellow) at Day 7 (D) and Day 14 (E). It reveals the complex vertical and lateral ingrowth of neurovascular networks that are typically missed by conventional 2D histology.

Neurovascular reconstruction during cutaneous wound healing is spatially heterogeneous, characterized by region‐dependent nerve fiber regrowth kinetics and depth‐dependent predominance of angiogenesis. This complexity is poorly resolved by conventional two‐dimensional histology, which could fail to capture the three‐dimensional coupling of nerves and vessels across tissue layers [42]. To overcome this fundamental limitation, we implemented an advanced tissue‐clearing and 3D imaging workflow (Figure 7A). Specifically, we adapted the iDISCO+ protocol—involving fixation, dehydration, delipidation, immunostaining, and refractive index matching—to render whole‐mount skin samples optically transparent (Figure 7B,C) [43]. This methodology preserves tissue integrity of full‐thickness skin, facilitating high‐fidelity volumetric imaging and reconstruction of the entire neurovascular network without mechanical sectioning. Light‐sheet fluorescence microscopy, combined with three‐dimensional maximum‐intensity projection and reconstruction, enables visualization of the entire cutaneous vascular and neural network (Figure 7D,E and Figure S27).

**FIGURE 8:**
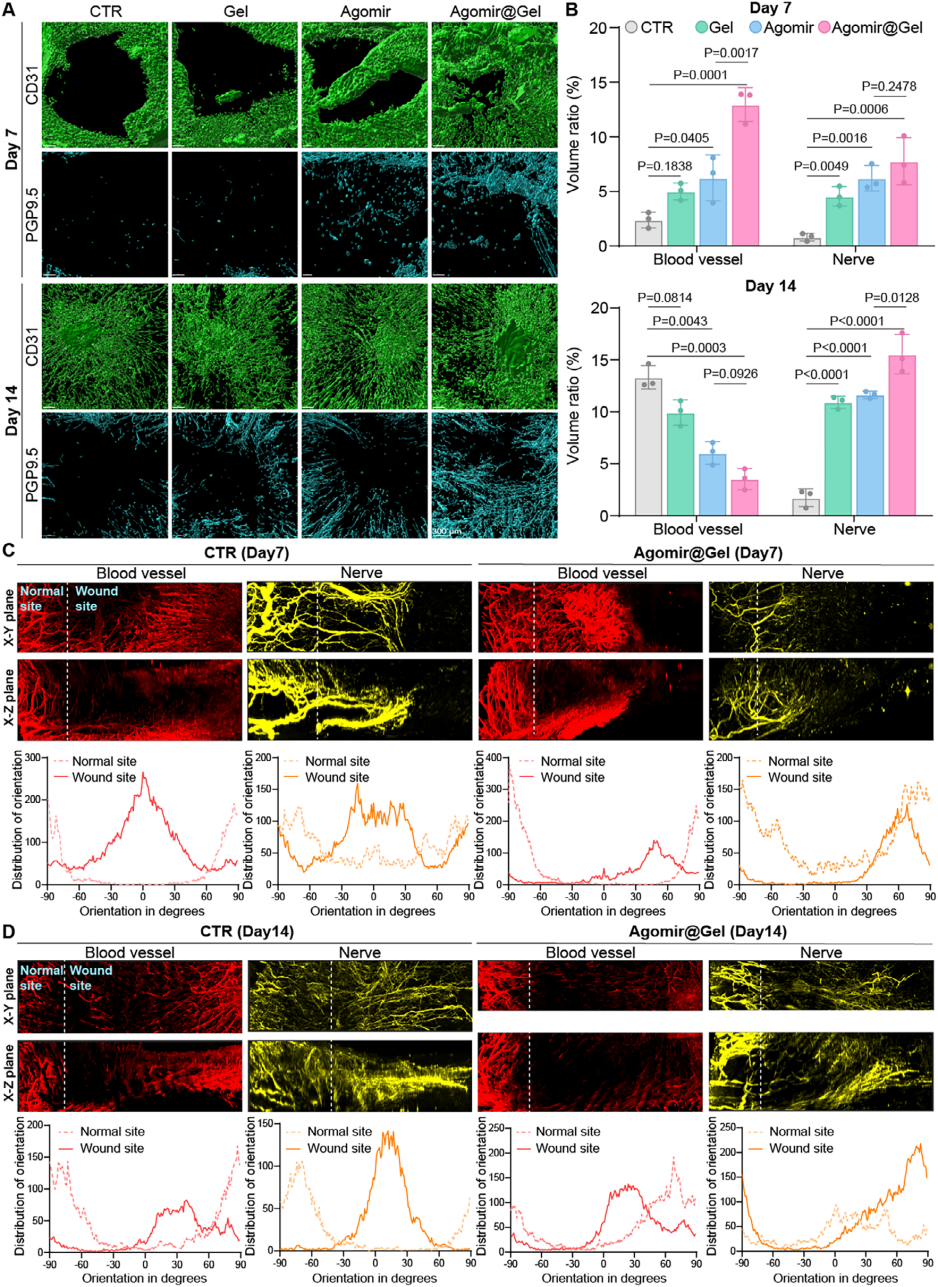
Spatiotemporal quantification confirms orchestrated neurovascular reconstruction in Agomir@Gel‐treated wounds. (A) High‐resolution surface‐rendered reconstructions of CD31^+^ vascular networks (green) and PGP9.5^+^ neural networks (blue) in the wound bed. (B) Volumetric quantification of blood vessels and nerves on Day 7 and 14. (C, D) Vector analysis of vascular and neural orientation angles and distribution histograms of orientation angles in normal skin versus the wound site, for control (CTR) and Agomir@Gel groups on day 7 (C) and day 14 (D). Normal site is shown on the left side of the white dashed line, with the wound site on the right. While vessels of wounds in the control group show disorganized, vertical alignment at the normal site and horizontal alignment at the wound site (indicative of immature sprouting), vessels in Agomir@Gel‐treated wounds show a coordinated transition from vertical slanted alignment, suggesting effective wound closure from the wound bed topside to bottom side. This slanted alignment of nerves in the wound sits is only observed in Agomir@Gel‐treated wounds, implying the enhanced spatiotemporal coupling of nerves and vessels.

It provided direct evidence that Agomir@Gel significantly enhanced the regeneration of 3D neurovascular networks. The wounds exhibited markedly elevated expression of the endothelial marker CD31, a marker of neovascularization. Compared with the blank CTR group (2.4%) and Agomir group (6.2%), the Agomir@Gel showed a significantly higher CD31‐positive area percentage of 13.0% on day 7. Subsequently, the regression of vascular networks was observed on Day 14, marking the onset of the tissue remodeling stage [44]. The volume ratio of vasculature networks in the Agomir@Gel group decreased to 3.5%, whereas that in the CTR group remained at 13.3%. This was accompanied by the formation of extensive and interconnected vascular networks in the Agomir@Gel groups. Interestingly, the neurogenesis visualized via pan‐neuronal marker PGP9.5‐positive nerve fibers appeared to lag behind the angiogenic process, yet persisted until Day 14, in contrast to the regressed vasculature. The Agomir@Gel group exhibited a significantly higher PGP9.5‐positive area percentage of 7.8% on day 7, compared to 0.8% in the CTR group. On day 14, the area percentage of PGP9.5‐positive nerve fibers reached 15.5% in the Agomir@Gel group, markedly higher than 1.7% in the CTR group (Figure 8A,B).

Furthermore, we observed significant spatial heterogeneity in the distribution and architecture of blood vessels and nerves between the normal and wound sites. Spatial mapping revealed the dynamic morphological evolution of regenerative signals, progressing from discrete spots at day 7 to continuous filamentous networks by day 14, both following defined pathways and infiltrating the target region primarily from its base and lateral aspects. Specifically, a key observation was the distinct spatial patterning of vasculature and innervation: transitioning from a vertical/multi‐angled architecture in normal tissue to a coordinated horizontal ingrowth within the wound bed. Compared to the CTR group, the Agomir@Gel group exhibited a reduced angle between the normal and wound tissue, suggesting a potential improvement in coordinating vascular and neural growth, which provided a key morphological insight for future studies on wound angiogenesis (Figure 8C,D and Movie S1–S4).

Collectively, these data illustrate a clear divergence in the spatio‐temporal coupling of neurovascular regeneration. This sequential pattern suggests that Agomir@Gel effectively coordinates the distinct early (angiogenic) and late (neurogenic) phases of tissue repair. This observed sequence aligns with established biological principles, as increasing evidence indicates that neurogenesis and angiogenesis are strongly related, with endothelial cells secreting factors that support neuronal growth and with neural precursor cells often proliferating in angiogenic environments [45].

## 3 Conclusions and Discussion

In summary, this study delineates a systemic “bone‐skin crosstalk” wherein fracture‐induced exosomal miR‐130b‐3p acts as a predominant regulator of cutaneous neurovascular coupling. By decoding this physiological repair mechanism, we successfully engineered a biomimetic therapeutic strategy that translates a systemic biological cue into a targeted, off‐the‐shelf nanomedicine. The development of cholesterol‐modified agomir‐130b‐3p enabled the spontaneous formation of stable, carrier‐free nanocomplexes, effectively overcoming the physiological barriers to miRNA delivery. Furthermore, encapsulating these nanocomplexes within a photocrosslinkable methacrylated collagen/silk fibroin hydrogel established a localized delivery depot that mimics the mechanical and biochemical cues of the native extracellular matrix.

Our implementation of advanced whole‐mount tissue clearing and volumetric imaging provided unique insights into the spatiotemporal dynamics of repair, revealing that the bio‐inspired nanoformulation not only accelerates wound closure but also guides the architectural reconstruction of deep vascular and neural networks. This work underscores the potential of exploiting endogenous inter‐organ signaling pathways to inform the design of next‐generation biomaterials. The agomir‐hydrogel nanoformulation presented here offers a scalable, non‐invasive alternative to surgical bone remodeling therapies, holding significant promise for the clinical management of refractory diabetic wounds and potentially other ischemic neurovascular conditions. Future studies may further explore the versatility of this strategy in delivering multiplexed signaling factors to resolve complex tissue defects.

Despite the promising therapeutic implications and mechanistic insights provided by this study, several limitations should be acknowledged.

First, at the tissue distribution level, the preferential accumulation of circulating fracture‐derived exosomes in distal wound sites may be influenced by the pathological microenvironment of diabetic wounds. Increased vascular permeability and endothelial barrier disruption could contribute to a phenomenon similar to the enhanced permeability and retention (EPR)‐like effect, thereby promoting passive extravasation and local retention of circulating nanoscale vesicles within injured tissues. However, given the complexity of wound pathophysiology, the relative contributions of passive leakage, impaired clearance, and potential cell‐type‐specific uptake mechanisms remain to be fully defined.

Second, at the molecular level, the intracellular regulatory mechanisms underlying the biological activity of miR‐130b‐3p warrant further investigation. SOCS5 was identified and validated as a direct downstream target of miR‐130b‐3p, suggesting its involvement as a key functional mediator in regulating endothelial and Schwann cell responses during neurovascular regeneration. Nevertheless, in line with the pleiotropic nature of miRNA‐mediated regulation, SOCS5 likely represents only one component of a broader regulatory network, and additional yet‐unidentified targets may also contribute to the observed biological effects.

Third, although fracture‐derived exosomes were identified as key mediators of bone‐skin crosstalk, their precise cellular origin within the fracture callus remains to be fully elucidated. The fracture callus constitutes a highly heterogeneous regenerative microenvironment composed of osteoblasts, osteoclasts, chondrocytes, endothelial cells, and various immune cell populations. A previous study investigating bone transport‐induced tissue regeneration suggested that osteoblasts are a major source of bone‐derived pro‐regenerative exosomes. Consistent with this notion, we observed substantial colocalization of exosomal signals with osteocalcin (OCN)‐positive cells within the fracture callus, suggesting that osteoblast‐lineage cells may represent a predominant source of the miR‐130b‐3p‐enriched exosomes identified in this study. Nevertheless, contributions from other resident or infiltrating cell populations cannot be excluded. Future studies employing lineage‐specific tracing and cell‐type‐resolved analyses will be required to definitively determine the cellular origin of fracture‐derived exosomes and their respective roles in mediating systemic regenerative signaling.

Fourth, although Cy3 fluorescence was used to monitor the in vivo release profile of Agomir@Gel, fluorescence signals alone cannot directly confirm the structural integrity of the released agomir in the protease‐ and nuclease‐rich microenvironment of diabetic wounds. While direct physicochemical characterization of agomir integrity in situ remains technically challenging, our qPCR‐based analysis demonstrated sustained and robust upregulation of miR‐130b‐3p in wound tissues over the 14‐day period following a single administration of Agomir@Gel. Notably, this level of miR‐130b‐3p elevation was consistently higher than that achieved by repeated administration of free agomir at corresponding time points. These findings provide functional evidence that the released agomir remains bioavailable and retains biological activity in vivo. Nevertheless, we acknowledge that this does not directly exclude the possibility of partial degradation of the oligonucleotide during sustained release, and future studies employing more direct analytical approaches will be required to further assess the molecular stability of agomir within the hydrogel system and wound microenvironment.

Fifth, as this study was primarily designed as a mechanistic and proof‐of‐concept investigation, the sample sizes remained relatively limited and were not based on formal power calculations. Future studies with larger cohorts and expanded validation will further strengthen the statistical robustness and translational relevance of these findings.

## 4 Methods

### 4.1 Animal Models

Male C57BL/6J mice (RRID: IMSR_JAX:000664) were obtained from GemPharmatech Co., Ltd. and maintained under specific pathogen‐free conditions with ad libitum access to food and water, a 12 h light–dark cycle, and controlled temperature and humidity. No animal exhibited a body weight loss exceeding 15% of its initial body weight at any time during the study (Figure S28).

To establish a type 1 diabetes mellitus model, 8‐week‐old male mice were injected intraperitoneally with streptozotocin (STZ, 40 mg/kg, 60256ES80; Yeasen, Shanghai, China) for 5 consecutive days, as per established protocols [46]. Four weeks after the final injection, fasting blood glucose levels were measured from tail vein samples, and mice with glucose levels exceeding 11.1 mmol/L were considered diabetic and included in subsequent experiments.

To generate a bone fracture model, fracture‐healing studies were performed using a semi‐stabilized femoral fracture approach as previously described [47]. Briefly, mice were anesthetized, and the distal femur was exposed to access the intramedullary canal. A guidewire was inserted to facilitate stabilization, and a controlled mid‐diaphyseal fracture was created. A stainless‐steel pin was then positioned over the guidewire to stabilize the fracture, after which the guidewire was removed and the incision sutured. Postoperative radiographs were obtained to confirm pin placement and stability.

To generate a full‐thickness excisional wound, the dorsal hair was removed, and the skin was disinfected. An 8 mm circular wound was created using a sterile biopsy punch, excising tissue down to the panniculus carnosus, and covered with a 3 M Tegaderm transparent film dressing (1622 W) to allow healing by secondary intention.

All animal experiments were conducted in accordance with institutional guidelines for animal care and use and were approved by the Animal Ethics Committee of Nanjing Drum Tower Hospital, the Affiliated Hospital of Nanjing University Medical School. (Approval No. AE01036). Animals were monitored daily throughout the experimental period, and tissues were harvested at designated time points for further analyses.

### 4.2 Wound Closure Analysis

Digital photographs of wounds were acquired at the indicated time points, and wound areas were quantified using ImageJ software. For each mouse, the wound area measured immediately after surgery (Day 0) was defined as 100%. Wound closure was normalized to the initial wound size of the corresponding animal and calculated using the following equation: Wound closure rate (%) = [(A_0_ − A_t_) / A_0_] × 100, where A_0_ represents the initial wound area at Day 0, and A_t_ represents the wound area at the indicated time point.

### 4.3 Small RNA Sequencing and Bioinformatic Analysis

Exosomes were isolated from serum as described earlier. Total RNA was extracted using RNAiso Plus following the manufacturer's instructions. RNA quality and quantity were assessed to confirm suitability for sequencing. Small RNA libraries were prepared and sequenced by HaploX Medical Laboratory Co., Ltd. (Jiangxi, China) on the Illumina platform. Differentially expressed small RNAs (DEsmallRNAs) were identified with a threshold of log_2_ fold change >1 and adjusted *p* < 0.05. Target genes of the DEsmallRNAs were predicted, and functional enrichment analysis was performed using Gene Ontology (GO) and Kyoto Encyclopedia of Genes and Genomes (KEGG) pathways in R. These analyses identified key biological processes and signaling pathways linked to the exosomal small RNAs.

### 4.4 Characterization of Agomir Structure and Endocytic Pathways

Agomir and Cyanine3‐labeled Agomir (Agomir‐Cy3) were ordered from Kileafbio Co., Ltd. (Nanjing, China). The hydrodynamic diameter of the nanoparticles was measured by dynamic light scattering (DLS). The agomir sample was prepared as a suspension in phosphate‐buffered saline (PBS) at a concentration of 2 nmol/mL. To eliminate dust and large aggregates, the suspension was filtered through a 0.22 µm syringe filter immediately before analysis. DLS measurements were conducted using a Malvern Zetasizer Pro (Malvern Panalytical, UK) at 25°C. The mean hydrodynamic diameter was derived from the analysis using the instrument's proprietary software.

Purified agomir was prepared for transmission electron microscopy (TEM) using phosphotungstic acid (PTA) negative staining. Briefly, a 10 µL aliquot of the agomir solution was applied onto a glow‐discharged carbon‐coated copper grid and allowed to adsorb for 10 min. Excess liquid was carefully removed with filter paper. The grid was then stained by applying a 10 µL droplet of 2% (w/v) phosphotungstic acid solution (pH 7.0, R20745, Yuanye, Shanghai, China) for 1 min. After staining, the excess stain was immediately blotted away, and the grid was air‐dried completely. The samples were observed using a transmission electron microscope (HT7800, Hitachi, Japan) at 80 kV.

To investigate the phagocytic uptake and intracellular trafficking of agomir, HEUVCs and SCs were seeded onto glass‐bottom confocal dishes (1051001, Zhejiang Saining Biotechnology Co., Ltd., China). For live‐cell lysosomal labeling, the LysoTracker (1:10000, A66438, Thermo Fisher, USA) working solution was added and incubated for 1 min at 37°C; the cells were then immediately washed with fresh medium. Next, Agomir‐Cy3 or Minic‐Cy3 (0.1 nmol/mL) was directly applied to the cells, and a spinning‐disk confocal microscope (Spin SR, Olympus, Japan) and a dual‐modality super‐resolution microscope (Cell Xpanse, CSR Biotech, Guangzhou, China) were used to acquire high‐resolution time‐series images.

To determine the cellular internalization mechanism of cholesterol‐modified agomir nanoparticles, we performed pharmacological inhibition assays using established endocytic pathway inhibitors. Briefly, cells were pre‐incubated with the following inhibitors for 30 min at 37°C before treatment with Agomir‐Cy3: EIPA (5‐(N‐ethyl‐N‐isopropyl)amiloride) (25 µM, HY‐101840, MCE, USA), Chlorpromazine (CPZ) (10 µM, HY‐12708, MCE, USA), Nystatin (10 mM, HY‐17409, MCE, USA), Methyl‐β‐cyclodextrin (MCD) (250 µM, HY‐101461, MCE, USA). Next, Agomir‐Cy3 (0.1 nmol/mL) was directly applied to the cells and incubated for 1 h, a dual‐modality super‐resolution microscope (Cell Xpanse, CSR Biotech, Guangzhou, China) were used to acquire high‐resolution images. Quantification was performed based on the percentage of intracellular fluorescence.

### 4.5 Tissue Clearing and 3D Imaging

The iDISCO+ protocol was implemented with minor modifications based on established methods. Briefly, fixed skin tissue samples were dehydrated through a graded methanol (10014118, Sinopharm, Shanghai, China)/water series (20%, 40%, 60%, 80%, and 100% methanol; 1 h per step at room temperature with gentle shaking). Subsequently, samples were delipidated overnight at room temperature in a 1:2 (v/v) mixture of methanol and dichloromethane (DCM, 80047318, Sinopharm, Shanghai, China). Following delipidation, samples were rehydrated in a descending methanol series (100%, 80%, 60%, 40%, and 20% methanol; 1 h per step) and then washed twice with PBS.

For whole‐mount immunostaining, samples were permeabilized and blocked overnight at 37°C in PBS containing 0.5% Triton X‐100 (T8200, Solarbio, Beijing, China), 10% dimethyl sulfoxide (DMSO, 40018269, Sinopharm, Shanghai, China), and 10% normal donkey serum (SL050, Solarbio, Beijing, China). The samples were then incubated with primary antibodies (CD31/PECAM‐1 Antibody, RRID: AB_2161028, AF3628, R&D, USA, and UCH‐L1/PGP9.5 Polyclonal antibody, RRID: AB_2210497, 14730‐1‐AP, Proteintech, USA, both at 1:400 dilution) in PBS containing 0.5% Triton X‐100, 10% DMSO, and 2% donkey serum at 37°C for 4 days with gentle shaking. After extensive washing with PBS containing 0.5% Triton X‐100 and 2% donkey serum at 37°C for 1 day, samples were incubated with secondary antibodies conjugated to Alexa Fluor 488 or 647 (RRID: AB_2687506 (ab150129), RRID: AB_2752244 (ab150075), Abcam, USA, 1:400 dilution) under the same conditions for an additional 4 days. Finally, samples underwent another extensive washing step at 37°C for 1 day.

Following immunostaining, samples were processed for clearing. Briefly, they were dehydrated again through a graded methanol series (40%, 80%, and 100% methanol; 1 h per step at room temperature with gentle shaking). Subsequently, samples were delipidated in a 1:2 (v/v) mixture of methanol and DCM for 3 h at room temperature. After two 15‐minute washes in 100% DCM, samples were incubated in dibenzyl ether (D107584, Aladdin, Shanghai, China) for refractive index matching until they became optically transparent. All steps from dehydration to clearing were performed in light‐protected glass vials. The cleared samples were imaged using a light‐sheet fluorescence microscope (LiTone XL2, Light Innovation Technology, China) equipped with a 5x objective lens (NA = 0.35). The raw image data (TIFF format) were first stitched and converted using LitScan 3.3.1. Subsequently, the processed volumetric datasets were imported into Imaris 10.2 software (Oxford Instruments) for 3D visualization, reconstruction, and quantitative analysis. Nerve fibers and blood vessels were segmented using absolute intensity‐based surface reconstruction. Segmentation thresholds were optimized for each immunohistochemical marker and maintained consistently across all experimental groups. From this initial segmentation, volumetric data for the PGP9.5‐positive and CD31‐positive structures were exported. Finally, volume density was calculated by normalizing the total volume of nerves or blood vessels to the volume of the region of interest (VOI).

## Author Contributions

T. S., H. S., and P. C. conceived and conceptualized the project. T.S. and H.S. obtained the data and wrote the manuscript. W. W. performed data analysis. Z. J. assisted with data analysis and animal experiments. B. L., Y. X., and R. P. performed cell‐based experiments. A. Y. performed light‐sheet microscopy imaging and related data analysis. Y. B., Y. S., and X. C. conducted animal experiments. B. G., Q. J., W. W., and P. C. supervised the project and revised the manuscript. All authors discussed the results and approved the final manuscript.

## Conflicts of Interest

The authors declare no conflicts of interest.

## Supporting information




**Supporting File 1**: advs76500‐sup‐0001‐SuppMat.docx.

**Supporting File 2**: advs76500‐sup‐0002‐MovieS1‐S4.zip.

## Data Availability

The data that support the findings of this study are available on request from the corresponding author. The data are not publicly available due to privacy or ethical restrictions.
