# Supplementary material for: Unraveling Bone‐Skin Crosstalk Enables miRNA Nanoformulation for Cutaneous Neurovascular Reconstruction in Diabetic Mice
Source: Adv Sci (Weinh). 2026 Jul 11;13(55):e76500. doi: 10.1002/advs.76500 (PMC13355937; doi:10.1002/advs.76500)
Supplement: Supplementary file 1 [file ADVS-13-e76500-s002.docx]

**Supplementary Information**

Unraveling Bone-skin Crosstalk Enables miRNA Nanoformulation for Cutaneous Neurovascular Reconstruction in Diabetic Mice

*Tao Shen^‡^, Hongdou Shen^‡^, Weiyue Wu, Zhuoying Jiang, Bin Liu, Yansi Xian, Rui Peng, An Yan, Yu Ben, Xiang Chen, Baosheng Guo, ^*^ Qing Jiang, ^*^ Weijun Wang, ^*^ Pingqiang Cai^*^*

1. Division of Sports Medicine and Adult Reconstructive Surgery, Department of Orthopedic Surgery, Nanjing Drum Tower Hospital, Affiliated Hospital of Medical School, Nanjing University, 321 Zhongshan Road, Nanjing 210008, Jiangsu, PR China.

2. Chemistry and Biomedicine Innovation Center, Nanjing University, Nanjing 210093, P. R. China.

3. Branch of National Clinical Research Center for Orthopedics, Sports Medicine and Rehabilitation, PR China.

4. Co-innovation Center of Neuroregeneration, Nantong University, 19 Qixiu Road, Nantong 226001, PR China.

METHODS

**Cell lines and cell culture**

Human umbilical vein endothelial cells (HUVECs) were obtained from Procell Life Science & Technology Co., Ltd. (PUMC-HUVEC-T1, CL-0675). Cell identity was authenticated by short tandem repeat (STR) profiling, showing 100% concordance with the Cell Resource Identification Database (CCRID, http://www.cellresource.cn). Primary Schwann cells were isolated from the epineurium of 8–12-week-old male mice as previously described.^1^ Primary human dermal fibroblasts were obtained from surgical waste skin samples in accordance with previously published protocols.^2^

HUVECs were cultured in PUMC-HUVEC-T1 Cell Complete Medium (CM-0675, Procell). Primary Schwann cells were cultured in mouse Schwann cell complete culture medium (PCM-M-165, Zqxzbio, Shanghai, China). Primary human dermal fibroblasts were cultured in human dermal fibroblast complete culture medium (PCM-H-086, Zqxzbio, Shanghai, China). All cells were maintained at 37 °C in a humidified incubator containing 5% CO₂.

**Hematoxylin–eosin (HE) and Masson’s trichrome staining**

Skin samples were fixed in 4% paraformaldehyde (PFA, G1101, Servicebio, Wuhan, China) at room temperature overnight. After fixation, tissues were dehydrated through a graded ethanol (10009218, Sinopharm, Shanghai, China) series, cleared in xylene (10023418, Sinopharm, Shanghai, China), and embedded in paraffin. Paraffin blocks were sectioned at 5 μm thickness. Sections were deparaffinized, rehydrated through descending ethanol concentrations, and stained with hematoxylin and eosin (H&E, G1120, Solarbio, Beijing, China) to assess overall tissue morphology. Adjacent sections were stained with Masson’s trichrome using a Masson’s Trichrome Stain Kit (G13340, Solarbio, Beijing, China) for evaluation of collagen deposition. All stained sections were examined and imaged under a light microscope.

**Immunofluorescence staining**

Skin sections were deparaffinized and rehydrated through a graded ethanol series. Fracture callus samples were fixed in 4% paraformaldehyde and decalcified in 10% EDTA (pH 7.4) prior to paraffin embedding. Sections were deparaffinized and rehydrated through a graded ethanol series. Endogenous peroxidase activity was first quenched by incubation with 3% hydrogen peroxide (10011218, Sinopharm, Shanghai, China). Antigen retrieval was subsequently performed using Citrate Antigen Retrieval Solution (P0081, Beyotime, Shanghai, China). Sections were then permeabilized with Immunostaining Permeabilization Buffer containing Triton X-100 (P0096, Beyotime, Shanghai, China) and blocked with the appropriate serum to minimize nonspecific binding. Sections were incubated with primary antibodies diluted 1:200, followed by fluorophore-conjugated secondary antibodies diluted 1:1000. Nuclei were stained with 4′,6-diamidino-2-phenylindole (DAPI, C1002, Beyotime, Shanghai, China). Primary antibodies used included α-SMA (RRID: AB_2918504, 67735-1-Ig, Proteintech, USA), CD31 (RRID: AB_2881055, 28083-1-AP, Proteintech, USA), PGP9.5 (RRID: AB_2210497, 14730-1-AP, Proteintech, USA), CD63 (RRID: AB_2862515, A19023, Abclonal, Wuhan, China) and osteocalcin (G-5) (RRID: AB_10859392, sc-365797, Santa Cruz Biotechnology Inc.). Fluorophore-conjugated secondary antibodies comprised Donkey anti-Mouse IgG (H+L), Alexa Fluor™ 488 (RRID: AB_141607, A-21202, Invitrogen, USA), Donkey anti-Rabbit IgG (H+L), Alexa Fluor™ Plus 594 (RRID: AB_2762827, A32754, Invitrogen, USA), Donkey anti-Rabbit IgG (H+L), Alexa Fluor™ Plus 488(RRID: AB_2866495, A32790, Invitrogen, USA), and Donkey anti-Mouse IgG (H+L), Alexa Fluor™ Plus 647(RRID: AB_2866494, A32787, Invitrogen, USA).

**RNA sequencing and bioinformatic analysis**

Bone callus tissues were collected from mice at 7 days following fracture, with intact bone samples obtained from sham-operated controls (n = 3 per group). Immediately after collection, tissues were snap-frozen in liquid nitrogen and mechanically ground into a fine powder. Total RNA was isolated using RNAiso Plus (9109, Takara, Dalian, China), with ultrasonication applied to facilitate tissue disruption in accordance with the manufacturer’s protocol. RNA integrity and purity were evaluated before library preparation. Sequencing libraries were constructed and subjected to high-throughput sequencing by HaploX Medical Laboratory Co., Ltd. (Jiangxi, China) on an Illumina platform using paired-end 150 bp reads (PE150). Following quality control, clean reads were generated and used for transcript quantification and expression profiling. Differential expression analysis was performed to identify genes exhibiting significant changes, using cutoffs of log₂ fold change > 1 and adjusted p < 0.05. Gene Ontology (GO), Kyoto Encyclopedia of Genes and Genomes (KEGG), and Gene Set Enrichment Analysis (GSEA) were conducted in R to characterize biological processes and signaling pathways associated with fracture-related callus formation.

**Exosome Isolation and Characterization**

Blood was collected from anesthetized mice at 7 days post-fracture and age-matched sham-operated controls (n = 4 per group). After clotting, serum was separated by centrifugation at 3000 x g for 10 minutes. Exosomes were isolated using the ExoQuick ULTRA EV Isolation Kit for Serum and Plasma (EQULTRA-20A-1, Systembio, USA) following the manufacturer’s instructions. For callus-derived EV isolation, fresh fracture callus tissues were harvested from mice at the indicated time points after femoral fracture. The tissues were thoroughly rinsed with ice-cold PBS and carefully trimmed with a sterile scalpel to remove adherent skeletal muscle and residual blood contamination. The callus tissues were then minced into small fragments and transferred into centrifuge tubes containing digestion buffer composed of collagenase D (2 mg/mL) and DNase I (40 U/mL). Tissue digestion was performed at 37°C for 60 min under gentle agitation. Following digestion, the tissue suspension was filtered through a sterile 70-μm cell strainer into a 50-mL centrifuge tube to remove undigested tissue fragments. The remaining tissue fragments were washed with PBS, and the wash solution was passed through the same strainer to maximize EV recovery. The filtrate was sequentially centrifuged at 300 × g for 10 min and 2,000 × g for 20 min at 4°C to remove cells and tissue debris. The resulting supernatant was further centrifuged at 10,000 × g for 30 min at 4°C to eliminate large vesicles, apoptotic bodies, and organelle fragments. Subsequently, the supernatant was filtered through a 0.22-μm membrane filter and ultracentrifuged at 100,000 × g for 70 min at 4°C using an Optima L-80 XP ultracentrifuge equipped with a Type 70 Ti rotor (Beckman Coulter, Brea, CA, USA). The EV pellet was washed once with sterile PBS and subjected to a second ultracentrifugation step at 100,000 × g for an additional 70 min. The final EV pellet was resuspended in PBS and either used immediately for downstream experiments or stored at −80°C until further analysis.

Exosomal proteins were then analyzed by Western blot to verify the presence of exosome markers. Exosome concentration and size distribution were determined via nanoparticle tracking analysis (NTA). Morphological characteristics of the exosomes were observed using transmission electron microscopy (TEM).

**RNA Extraction and qPCR Analysis**

Total RNA was extracted from bone and skin tissues using RNAiso Plus and from cells using the Super FastPure Cell RNA Isolation Kit (RC102, Vazyme, Nanjing, China), according to the manufacturer’s protocol. cDNA synthesis was carried out with the HiScript IV All-in-One Ultra RT SuperMix for qPCR (R433, Vazyme, Nanjing, China), following the manufacturer’s instructions. Quantitative PCR was performed using the SupRealQ Ultra Hunter SYBR qPCR Master Mix (Q713, Vazyme, Nanjing, China.) on a QuantStudio 5 Real-Time PCR System (Thermo Fisher, USA). Gene expression was analyzed using the comparative CT method. Socs5 expression was normalized to Actb as the internal control. For miR-130b-3p, the stem-loop method was employed for amplification. Expression levels of miR-130b-3p were normalized to U6 as the internal control, and CEL-67 served as the external control for miRNA quantification. Stem-loop RT primers and qPCR primers for miR-130b-3p, U6, and Cel-67 were synthesized and supplied by Kileafbio Co., Ltd. (Nanjing, China). Socs5 and Actb primer sequences used are listed below: Socs5, Forward: 5’-GTGCCACAGAAATCCCTCAAA, Reverse: 5’-TCTCTTCGTGCAAGTCTTGTTC; Actb: 5’-GGCTGTATTCCCCTCCATCG, Reverse: 5’-CCAGTTGGTAACAATGCCATGT.

**Cell Viability Assay**

Cell viability was assessed using the Cell Counting Kit-8 (HY-K0301, MedChemExpress, USA), according to the manufacturer’s instructions. Cells were seeded in 96-well plates at a density of 5 × 10⁴ cells per well and incubated overnight. After transfection with miR-130b-3p, the medium was replaced, and the cells were incubated for an additional 24 hours. Then, 10 μL of CCK-8 solution was added to each well, and the cells were incubated for 2 hours at 37 ºC. Absorbance at 450 nm was measured using a microplate reader. Cell viability was calculated as a percentage of the control group, which was set to 100%.

**miR-130b-3p and SOCS5 Overexpression Plasmid Transfection**

MiR-130b-3p mimic and SOCS5 overexpression plasmid were transfected into cells using Lipofectamine™ 3000 Transfection Reagent (L3000075, Invitrogen, USA) according to the manufacturer’s protocol. Cells were seeded in 6-well plates at a density of 1 × 10⁵ cells per well and cultured overnight. The following day, cells were transfected with 100 nmol of miR-130b-3p mimic (HanBio Technology, Shanghai, China) or 2.5 μg of SOCS5 plasmid (OBiO Technology, Shanghai, China) in serum-free medium. The transfection complexes were prepared by combining the mimic or plasmid with Lipofectamine™ 3000 reagent, incubating for 15 minutes at room temperature. The mixture was then added to the cells, which were incubated for 6–8 hours. After transfection, the medium was replaced with fresh complete medium, and cells were cultured for an additional 24 hours before further analysis.

**Western Blot**

Total protein was extracted from cells using the Total Protein Extraction Kit (KGB5303, KeygenBioTECH, Nanjing, China) following the manufacturer's protocol. Cells were lysed on ice for 15 minutes with intermittent vortexing, then detached from the culture plates using a cell scraper. The lysates were centrifuged at 12,000 × g for 15 minutes at 4 ºC, and the supernatant was collected. Protein samples were mixed with 5 × SDS-PAGE loading buffer (G2527, LABLEAD, Beijing, China) by vortexing with a vortex mixer and heated at 99 ºC for 10 minutes to denature. Proteins were separated by SDS-PAGE and transferred to a PVDF membrane (IPVH00010, Millipore, USA). The membrane was blocked with NcmBlot Blocking Buffer (P30500, New Cell & Molecular Biotech, Suzhou, China) at room temperature for 1 hour. After blocking, the membrane was incubated overnight at 4 ºC with the primary antibody (1:1000 dilution). Following incubation, the membrane was washed three times with Takara T. The membrane was then incubated with an HRP-conjugated secondary antibody (ZYID002-0050, Zunyan, Nanjing, China) at room temperature for 1 hour. After further washes with PBST, protein bands were detected using an enhanced chemiluminescence substrate (KF8001, Affinity, USA). Primary antibodies used included CD9 (104834-T32, Sino Biological, Beijing), CD63 (A19023, Abclonal, Wuhan, China), CD81 (RRID: AB_3070067, ET1611‐87, HUABIO, Hangzhou, China), TSG101 (RRID: AB_2863517, A5789, Abclonal, Wuhan, China), SOCS5 (A7952, Abclonal, Wuhan, China) and β‐actin (RRID: AB_2768234, AC026, Abclonal, Wuhan, China).

**Tube Formation Assay**

GelNest™ Basement Membrane Matrix (211252, NEST, Wuxi, China) was thawed on ice and briefly centrifuged to remove trapped air bubbles prior to use. The matrix was then aliquoted into a 96-well plate (50 µL per well) and allowed to solidify at 37 °C for 1 h. Following this, HUVECs were serum-starved for 12 hours to induce quiescence. After starvation, HUVECs were counted using the Countstar Mira BF-S (P020300026, Countstar, Shanghai, China), and the cell density was adjusted to 2 × 10^5^ cells/mL. A 50 µL aliquot of the cell suspension was added to each well containing the solidified matrix gel. The plate was incubated at 37 ºC for 6–12 hours, allowing the cells to form tubular structures.

**Cell Migration Assay**

SC cells and HDF cells were serum-starved for 12 hours. After starvation, the cells were digested with Trypsin EDTA Solution (A300-100, BDBIO, Shanghai, China) and resuspended in serum-free medium. The cell suspension was then added to the upper chamber of a Transwell insert (TCS003012, Jet Biofil, Guangzhou, China). In the lower chamber, medium containing 20% fetal bovine serum (FBS-303, Jin Yuan Kang Biotechnology, Inner Mongolia, China) was added as a chemoattractant. The cells were allowed to migrate for 24 hours at 37 ºC. After migration, the medium in both the upper and lower chambers was removed. The migrated cells on the lower surface of the membrane were fixed with 4% paraformaldehyde for 15 minutes, then washed with distilled water. Subsequently, the membrane was stained with crystal violet (C0121, Beyotime, Shanghai, China) for 10 minutes. After staining, the membrane was washed with distilled water to remove excess dye. Migrated cells were observed and quantified under a microscope.

**Endothelial Cell–Schwann Cell Co-culture Assays**

To evaluate the effects of miR-130b-3p on endothelial–Schwann cell crosstalk, co-culture systems were established using Transwell inserts.

For the co-culture tube formation assay, Schwann cells (SCs) transfected with miR-130b-3p mimic or negative control (NC) were seeded into the upper chamber of a Transwell insert (0.4 μm pore size), while HUVECs were seeded onto GelNest™ Basement Membrane Matrix-coated 12-well plates in the lower chamber. After co-culture for 6 h, tube-like structures formed by HUVECs were photographed under an inverted microscope and quantified using ImageJ software.

For the co-culture migration assay, HUVECs transfected with miR-130b-3p mimic or NC were seeded into the lower chamber of a 24-well Transwell system. Serum-starved SCs were seeded into the upper chamber (8 μm pore size) in serum-free medium. After incubation for 24 h, non-migrated cells on the upper surface of the membrane were removed with a cotton swab. Cells that migrated to the lower surface were fixed with 4% paraformaldehyde for 15 min and stained with crystal violet for 10 min. Migrated cells were photographed and quantified under a microscope.

**ELISA Analysis of Neurovascular Coupling-Related Factors**

To investigate the paracrine mechanisms underlying endothelial–Schwann cell communication, Schwann cells (SCs) or human umbilical vein endothelial cells (HUVECs) were transfected with agomir-130b-3p or negative control (NC). After 48 h of culture, conditioned media were collected and centrifuged at 3,000 rpm for 10 min to remove cell debris.

The concentrations of vascular endothelial growth factor (VEGF) and platelet-derived growth factor-BB (PDGF-BB) in SC-conditioned media, as well as nerve growth factor (NGF) and brain-derived neurotrophic factor (BDNF) in HUVEC-conditioned media, were quantified by enzyme-linked immunosorbent assay (ELISA). ELISA analyses were performed by UpingBio Technology Co., Ltd. (Hangzhou, China) according to the manufacturer's standard protocols. Cytokine concentrations were calculated based on standard curves.

**Preparation and Characterization of Methacrylated Collagen and Silk Fibroin.**

Male SD Rats (8-week-old) (RRID: MGI:5651135) were obtained from GemPharmatech Co., Ltd. Briefly, collagen type I was extracted from rat tails.^3^ 10 g tendons and 500 ml of 3% (v/v) glacial acetic acid (10000208, Sinopharm, Shanghai, China) were added at a stirring speed of 150 rpm for 36 h. Next, the solution was centrifuged at 10,000 rpm for 20 min to remove the residual impurities. Then, the solution was dialyzed against an acetic acid aqueous solution (pH 4) using dialysis tubing with 3500 Da MWCO ([MD3534](https://shyuanye.com/goods-MD3534.html), Yuanye, Shanghai, China) for 48 h; the final collagen concentration was 3–5 mg/ml. The methacrylated collagen (ColMA) was synthesized via a chemical grafting reaction.^4, 5^ Glycidyl methacrylate (G106686, Aladdin, Shanghai, China) was added to a collagen type I solution (pH 4) at a 1:1 mass feed ratio. The reaction mixture was then vigorously stirred at 37 ºC for 12 hours. Then, the solution was purified by dialysis against deionized water using dialysis tubing with 3500 Da for 5 days. Finally, the purified ColMA solution was lyophilized to obtain a dry, porous sponge, which was stored at −20 ºC until use.

Bombyx mori cocoons were supplied by sericulture practitioners (Xuzhou, China). Silk cocoon pieces (15 g) were degummed by boiling in 3 L of a 0.02 M Sodium carbonate ([10019260](https://www.reagent.com.cn/goodsDetail/Sodium-carbonate/%E7%A2%B3%E9%85%B8%E9%92%A0%EF%BC%8C%E6%97%A0%E6%B0%B4/e02827aa05244f12b586fe6c0d88959c), Sinopharm, Shanghai, China) solution at 100 ºC for 30 min to remove sericin. Subsequently, 2 g of the degummed silk fibroin (SF) were dissolved completely in 8 mL of a 9.3 M Lithium bromide (A11996, Energy Chemical, Shanghai, China) solution at 60 ºC for 4 h. The solution was dialyzed against distilled water for 48 h using dialysis tubing with 3500 Da. Next, the solution was centrifuged at 9,000 rpm for 15 min to eliminate any residual impurities. Finally, the purified SF aqueous solution was obtained and stored at 4 ºC for subsequent use^6^.

The successful methacrylation of collagen was confirmed by proton nuclear magnetic resonance spectroscopy (600MHz, Bruker, Germany). For the analysis, lyophilized samples of both native collagen and the synthesized ColMA were separately dissolved in deuterium oxide (E090001, Energy Chemical, Shanghai, China) containing 3% (v/v) acetic acid-d₄ (E090026, Energy Chemical, Shanghai, China) at a concentration of 10 mg/mL.

The lyophilized collagen and ColMA (1mg/ml) were denatured at 99 ºC for 10 min. Samples and pre-stained protein standards were separated by SDS-PAGE using an 10% resolving gel. The gel was stained with BeyoBlue^TM^ Plus Coomassie Blue Super Fast Staining Solution (P0003, Beyotime, Shanghai, China) for 15 min, followed by destaining in the same solution until bands were clear. Apparent molecular weights were estimated by comparing band migration distances to the standard curve.

**Preparation and Characterization of ColMA/SF Hydrogels.**

The lyophilized ColMA was first dissolved in 1× PBS under constant stirring at 37 °C to obtain a homogeneous precursor solution. Subsequently, to formulate the photocrosslinkable bioink, predetermined volumes of this ColMA stock solution (final concentration: 40 mg/mL), SF (0–20 mg/mL), and Lithium Phenyl (2,4,6-trimethylbenzoyl) phosphinate (0.04% w/v, L0290, TCI, Shanghai, China) were sequentially added and thoroughly mixed in 1× PBS. The mixture was exposed to ultraviolet (UV) light (365 nm) for 2 minutes using a UV curing lamp (SCIENTZ03-II, Ningbo, China).

Briefly, the ColMA/SF hydrogels were rapidly frozen in liquid nitrogen and then lyophilized to obtain dry scaffolds. Next, the samples were sputtered with a layer of gold at a current intensity of 15 mA for 30 s. The microstructural observation was then carried out on an environmental scanning electron microscope (Quattro S, Thermo Fisher, USA) at a voltage of 5 kV.

The hydrogels (diameter: 10 mm, height: 4 mm) were fabricated in a Teflon mould, and compression stress–strain curves were collected using an electronic universal testing machine (UTM 6502, Suns, Shenzhen, China) with 80% strain at a speed of 5 mm/min. The compression modulus was obtained by calculating the slope of the 5–15% strain. Sequential cyclic strain testing was conducted from 0–50% strain at a speed of 20 mm/min for 10 cycles.

To evaluate the mechanical stability and viscoelastic properties of the hydrogels, the storage modulus (G′) and loss modulus (G″) were measured using a Thermo-Haake rheometer (RS6000, Thermo Scientific, USA). Dynamic frequency sweeps were executed using parallel-plate geometry (diameter: 8mm, 0.5mm gap) from 10 to 1 rad/s at a constant shear stress of 1 Pa and a controlled temperature of 25 ºC.

The cytocompatibility of the hydrogels was assessed through direct contact culture combined with live/dead and proliferation assays. Briefly, uniform hydrogel discs (60 µL) were fabricated in a 48-well plate. After equilibration with culture medium, a suspension of 5,000 cells in 300 µL of complete medium was seeded onto each hydrogel surface and incubated for 2 days. For live/dead staining, hydrogel-cell constructs were rinsed with PBS and incubated with Calcein AM/PI Double Staining Kit(E-CK-A354, Elabscience Biotechnology Co., Ltd.) at 37 ºC for 30 min in the dark. For proliferation (EdU) staining, constructs were incubated with 10 µM EdU prepared using the EdU Imaging Kits (Cy5, K1076, Apexbio). Cells were then fixed with 4% paraformaldehyde (M40958, AbMole, USA), permeabilized with 0.3% Triton X-100, and incubated with the Click reaction cocktail for 30 min. Nuclei were counterstained with DAPI before imaging. An inverted fluorescence microscope (DMI8, Leica, Germany) was used to acquire cell images.

For the degradation test, 400 µL PBS was added to 200 µL of SF hydrogels and then incubated at 37 ºC for 1, 3, 5, 7, and 14 days. The PBS solution was changed every 2 days. SF hydrogels were rinsed twice with distilled water, lyophilized, and weighed.

The release kinetics of Agomir-Cy3 from the hydrogel were evaluated to characterize its sustained delivery potential, loaded with 2.5 nmol of Agomir-Cy3, was immersed in 300 µL of PBS buffer as the release medium. At predetermined intervals, the supernatant was withdrawn for analysis. The fluorescence intensity (excitation/emission: 555 nm/595 nm) of the released Agomir was quantified using a microplate reader (SpectraMax iD5, Molecular Devices, USA). The cumulative release percentage was calculated.

**Statistical analysis**
All quantitative data are presented as mean ± SD. Statistical analyses were performed using GraphPad Prism. For comparisons between two groups, an unpaired two-tailed Student’s t test was used. For comparisons among multiple groups, one-way analysis of variance (ANOVA) followed by Tukey’s multiple comparisons test was applied. ns indicates no statistical significance (P > 0.05).

**Figure S1. Femoral fracture enhances wound healing and collagen remodeling in diabetic mice.** (A) Representative H&E staining of wound sections at the indicated time points. Arrows denote the wound margins. Scale bar, 1 mm. (B) Quantification of collagen remodeling. Data are mean ± SD; n = 4 mice per group. (C) Representative Masson’s trichrome staining of wound sections at the indicated time points. Arrows denote the wound margins. Scale bar, 1 mm. (D) Summary schematic depicting the association between femoral fracture and enhanced wound repair in diabetic mice.

**Figure S2. Gene Ontology (GO) enrichment analysis of differentially expressed genes identified in fracture callus, categorized into biological process (BP), cellular component (CC), and molecular function (MF).**

**Figure S3. Colocalization of exosomal signals with osteoblast-lineage cells in the fracture callus**. Representative immunofluorescence images of fracture callus tissue showing staining for exosomal marker CD63 (green), osteocalcin (OCN, red), and nuclei (DAPI, blue). Scale bar = 100 μm.

**Figure S4. Identification of circulating exosomes and pharmacological modulation of exosome-related signals.** (A) Western blot analysis of canonical serum exosome markers (CD9, CD63, CD81, and TSG101). (B) Nanoparticle tracking analysis (NTA) of circulating exosomes isolated from the serum of fractured mice. (C) Immunofluorescence staining of CD63 in sham, bone fracture, and bone fracture+GW4869 groups; nuclei were counterstained with DAPI (blue). Scale bar, 100 μm.

**Figure S5. Isolation, characterization, and in vivo tracking of fracture-derived exosomes.** (A) Representative transmission electron microscopy (TEM) images of exosomes isolated from Sham and fracture (BF) groups, showing typical cup-shaped morphology. Scale bar = 100 nm. (B) Western blot analysis of exosomal markers, including CD9, CD63, CD81, and TSG101, confirming successful isolation of exosomes from Sham and BF samples. (C) Nanoparticle tracking analysis (NTA) showing size distribution profiles of exosomes derived from Sham and BF groups. (D) In vivo fluorescence imaging of DiD-labeled exosomes in diabetic mice with full-thickness dorsal skin wounds at 1 h, 6 h, and 24 h after intravenous injection. Mice injected with unlabeled exosomes served as blank controls. (E) Quantitative analysis of fluorescence intensity at the wound site corresponding to (D). Data are presented as mean ± SD (n = 4).

**Figure S6. Local administration of GW4869 near normal bone does not affect CD63 expression in dorsal skin.** (A) Representative immunofluorescence images of dorsal skin tissue showing CD63 (green) and nuclei (DAPI, blue) in the Sham group and GW4869-treated group. Scale bar = 100 μm. (B) Quantitative analysis of CD63-positive area in dorsal skin showing no significant difference. Data are presented as mean ± SD. ns, not significant.

**Figure S7. GW4869 impairs fracture-enhanced wound healing and tissue remodeling..** (A) Representative photographs of wounds and corresponding wound area traces. All photographs are presented in 1 cm × 1 cm frames. (B) Hematoxylin and eosin (H&E) staining of wound sections at the indicated time points. Arrows denote the wound margins. Scale bar, 1 mm. (C) Masson’s trichrome staining at the indicated time points. Arrows denote the wound margins. Scale bar, 1 mm. (D) Quantitative of collagen remodeling. Data are mean ± SD. n = 4 mice per group.

**Figure S8. Gene Ontology (GO), categorized into biological process (BP), cellular component (CC), and molecular function (MF), and Kyoto Encyclopedia of Genes and Genomes (KEGG) pathway enrichment analysis of differentially abundant exosomal small RNAs.**

**Figure S9. Validation of miR-130b-3p overexpression and its effects on cell proliferation in HUVECs and Schwann cells.** (A, B) Quantitative PCR (qPCR) analysis of miR-130b-3p expression in human umbilical vein endothelial cells (HUVECs) (A) and Schwann cells (SCs) (B) transfected with negative control (NC) or miR-130b-3p mimic. Data are mean ± SD. n = 3 independent biological replicates. (C, D) Cell Counting Kit-8 (CCK-8) assays evaluating the effects of miR-130b-3p mimic on cell proliferation in HUVECs (C) and SCs (D) transfected with NC or miR-130b-3p mimic. Data are mean ± SD. n = 3 independent biological replicates.

**Figure S10. miR-130b-3p promotes functional neurovascular coupling through bidirectional endothelial–Schwann cell communication.** (A) Representative images and (B) quantitative analysis of tube formation by HUVECs co-cultured with NC- or miR-130b-3p-transfected SCs. (E) Representative images and (F) quantitative analysis of SC migration induced by NC- or miR-130b-3p-transfected HUVECs. (C–D) ELISA quantification of VEGF and PDGF-BB secretion by SCs following miR-130b-3p transfection. (G–H) ELISA quantification of NGF and BDNF secretion by HUVECs following miR-130b-3p transfection. Data are presented as mean ± SD (n = 3 independent experiments).

**Figure S11. Validation of SOCS5 as a direct target of miR-130b-3p. (**A**)** qPCR analyses of Socs5 mRNA expression in HUVECs transfected with negative control (NC) or miR-130b-3p mimic. Data are mean ± SD. n = 3 independent biological replicates. (B) Western blot analyses of SOCS5 protein expression in HUVECs transfected with NC or miR-130b-3p mimic. **(**C**)** qPCR analyses of *Socs5* mRNA expression in SCs transfected with NC or miR-130b-3p mimic. Data are mean ± SD. n = 3 independent biological replicates. (D) Western blot analyses of SOCS5 protein expression in SCs transfected with NC or miR-130b-3p mimic.

**Figure S12. Western blot analysis of SOCS5 protein expression in HUVECs (A) and SCs (B) during functional assessment experiments.**

**Figure S13. Functional validation of miR-130b-3p in human dermal fibroblasts (HDFs).** (A) qPCR analysis of miR-130b-3p expression in HDFs transfected with NC or miR-130b-3p mimic. Data are mean ± SD. n = 3 independent biological replicates. (B) CCK-8 assays evaluating the effect of miR-130b-3p mimic on HDF proliferation. Data are mean ± SD. n = 3 independent biological replicates. (C) Representative tube formation assays of HUVECs transfected with NC or miR-130b-3p mimic. Scale bar, 100 μm. (D) Quantitative analysis of the number of cells migrating in HDFs transfected with NC or miR-130b-3p mimic. Data are mean ± SD. n = 3 independent biological replicates. (E) qPCR analysis of Socs5 protein expression in HDFs transfected with NC or miR-130b-3p mimic. Data are mean ± SD. n = 3 independent replicates. (F) Western blot analysis of SOCS5 protein expression in HDFs transfected with NC or miR-130b-3p mimic.

**Figure S14. Functional validation of miR-130b-3p-SOCS5 axis in human dermal fibroblasts (HDFs).** (A) Functional experiments in HDFs examining the effects of SOCS5 overexpression on miR-130b-3p–regulated cell migration. Scale bar, 100 μm. (B) Quantitative analysis of the number of cells migrating in HDFs. Data are mean ± SD. n = 3 independent biological replicates. (C) Western blot analysis of SOCS5 protein expression in HDFs during functional experiments.

**Figure S15. RNase A degradation assay of agomir-130b-3p and unmodified miRNA mimic.**

**Figure S16. Zeta potential of agomir nanoassemblies measured by electrophoretic light scattering (ELS).**

**Figure S17.** **Photocrosslinkable collagen methacryloyl/silk fibroin hydrogel enables sustained local delivery of agomir-130b-3p.** (A) Schematic of the fabrication process of the collagen methacryloyl/silk fibroin (ColMA/SF) hydrogel loaded with agomir. (B) ^1^H Nuclear Magnetic Resonance spectrum (NMR) of native collagen confirming methacrylation. (C) Coomassie Brilliant Blue-stained SDS–PAGE comparing the molecular weight profiles of collagen and ColMA. (D) Representative photographs demonstrating conformal in situ gelation on defects of different geometries (circle, triangle, star) under torsion.

**Figure S18. Cytocompatibility assessment of HUVECs and Schwann cells on hydrogels surface with different SF concentrations**. (A) Representative fluorescence micrographs and quantification of HUVECs and Schwann cell cultured on hydrogel surfaces with varying SF concentration for 2 days, stained with Calcein-AM (green, live cells) and propidium iodide (PI, red, dead cells). (B) Representative fluorescence micrographs and quantification of HUVECs and Schwann cell cultured on hydrogel surfaces with varying SF concentration for 2 days, stained with 5-Ethynyl-2'-deoxyuridine (EDU, magenta, proliferating cells) and DAPI (blue, nuclei). Data are mean ± SD; n = 3 independent biological replicates.

**Figure S19. Characterization of photo-crosslinked ColMA_40_ and ColMA_40_SF_10_ hydrogels.** (A) Macroscopic appearance and scanning electron microscope (SEM) images of the photo-crosslinked ColMA_40_ and ColMA_40_SF_10_ hydrogels. (B) Cyclic compression stress-strain curves of the hydrogels. (C) Compressive modulus of the hydrogels. Data are mean ± SD. n = 3 independent replicates. (D) Frequency sweep rheology showing storage (G’) and loss (G’’) moduli.

**Figure S20. Cytocompatibility assessment of HUVECs and Schwann cells on hydrogels surface with and without agomir**. (A) Representative calcein-AM/PI live/dead staining and quantification of viable cells after 2-day culture on tissue-culture plastic (CTR), blank hydrogel (Gel; ColMA_40_SF_10_), hydrogel loaded with negative-control RNA (NC@Gel), or hydrogel loaded with agomir-130b-3p (Agomir@Gel) (0.5 nmol per gel). (B)Representative EdU/DAPI staining and quantification of EdU^+^ cells under the same conditions. Data are mean ± SD. n = 3 independent biological replicates.

**Figure S21. Cytocompatibility assessment of human dermal fibroblasts (HDFs) on hydrogel surfaces.** (A) Representative calcein-AM/PI live/dead staining and quantification of HDFs cultured for 2 days on hydrogels with varying silk fibroin (SF) concentrations, stained with Calcein-AM (green, live cells) and propidium iodide (PI, red, dead cells). CTR indicates standard microplate control. (B) Representative EdU/DAPI staining and quantification of EdU^+^ cells under the same conditions. (C) Representative calcein-AM/PI live/dead staining and quantification of viable cells after 2-day culture on tissue-culture plastic (CTR), blank hydrogel (Gel; ColMA_40_SF_10_), hydrogel loaded with negative-control RNA (NC@Gel), or hydrogel loaded with agomir-130b-3p (Agomir@Gel) (0.5 nmol per gel). (D) Representative EdU/DAPI staining and quantification of EdU^+^ cells under the same conditions. Data are mean ± SD. n = 3 independent biological replicates.

**Figure S22.** **Assessment of hydrogel degradation and in vitro release of the agomir.** (A) Cumulative release profile of agomir-130b-3p from ColMA_40_SF_10_ hydrogels within 14 days. (B) Fluorescent imaging of wound sections showing retention of Cy3-labeled agomir delivered in solution (Agomir) or via hydrogel (Agomir@Gel) over 14 days.

**Figure S23. Evaluation of the antioxidant capacity of the hydrogel.** Data are presented as mean ± SD, n = 3.

**Figure S24. Evaluation of the in vivo degradation behavior of the hydrogel.** (A) Representative in vivo fluorescence images showing the retention of the hydrogel in the wound at designated time points. (B) Quantitative analysis of the residual fluorescence intensity ratio over time. Data are presented as mean ± SD, n = 3.

**Figure S25. H&E staining of major organs (heart, liver, spleen, lung, and kidney) to assess biocompatibility.**

**Figure S26. Serum biochemical analysis for biosafety evaluation after treatment.** Serum levels of alanine aminotransferase (ALT), aspartate aminotransferase (AST), creatinine (CREA), and urea (UREA) were measured to assess potential hepatic and renal toxicity. Data are presented as mean ± SD (n = 4).

**Figure S27. Representative 3D view of the full-thickness wound bed stained for CD31 (vasculature, red) and PGP9.5 (nerves, yellow) at Day 7 (D) and Day 14.**

**Figure S28. Body weight monitoring during animal studies. Percentage body weight loss relative to the initial body weight was recorded throughout the experimental period.** (A) Body weight loss of mice in the Sham and BF groups corresponding to the experiments presented in Figure 2. (B) Body weight loss of mice in the Sham, BF, and BF + GW4869 groups corresponding to the experiments presented in Figure 2. (C) Body weight loss of mice in the Control, Gel, Agomir, and Agomir@Gel groups corresponding to the experiments presented in Figure 5. (D) Body weight loss of mice in the Control, Gel, Agomir, and Agomir@Gel groups corresponding to the experiments presented in Figure 7. Data are presented as percentage body weight loss relative to the initial body weight.

REFERENCES

(1) Huang, Y.; Wu, L.; Zhao, Y.; et al. Schwann Cell Promotes Macrophage Recruitment through Il-17b/Il-17rb Pathway in Injured Peripheral Nerves. *Cell Rep.* **2024**, *43* (2). 113753.

(2) Rnjak, J.; Li, Z.; Maitz, P. K. M.; et al. Primary Human Dermal Fibroblast Interactions with Open Weave Three-Dimensional Scaffolds Prepared from Synthetic Human Elastin. *Biomaterials* **2009**, *30* (32). 6469-6477.

(3) Rajan, N.; Habermehl, J.; Coté, M.-F.; et al. Preparation of Ready-to-Use, Storable and Reconstituted Type I Collagen from Rat Tail Tendon for Tissue Engineering Applications. *Nat. Protoc.* **2006**, *1* (6). 2753-2758.

(4) Drzewiecki, K. E.; Parmar, A. S.; Gaudet, I. D.; et al. Methacrylation Induces Rapid, Temperature-Dependent, Reversible Self-Assembly of Type-I Collagen. *Langmuir* **2014**, *30* (37). 11204-11211.

(5) Sk, M. M.; Das, P.; Panwar, A.; et al. Synthesis and Characterization of Site-Selective Photo-Crosslinkable Glycidyl Methacrylate Functionalized Gelatin-Based 3d Hydrogel Scaffold for Liver Tissue Engineering. *Mater. Sci. Eng. C Mater. Biol. Appl.* **2021**, *123*. 111694.

(6) Rockwood, D. N.; Preda, R. C.; Yücel, T.; et al. Materials Fabrication from Bombyx Mori Silk Fibroin. *Nat. Protoc.* **2011**, *6* (10). 1612-1631.
